# Bio-organic fertilizers improve *Dendrocalamus farinosus* growth by remolding the soil microbiome and metabolome

**DOI:** 10.3389/fmicb.2023.1117355

**Published:** 2023-02-15

**Authors:** Shangmeng Li, Wei Fan, Gang Xu, Ying Cao, Xin Zhao, Suwei Hao, Bin Deng, Siyuan Ren, Shanglian Hu

**Affiliations:** ^1^Lab of Plant Cell Engineering, Southwest University of Science and Technology, Mianyang, China; ^2^Engineering Research Center for Biomass Resource Utilizaiton and Modification of Sichuan Province, Mianyang, China

**Keywords:** *Dendrocalamus farinosus*, organic and microbial fertilizers, fertilization, soil microbiome, bacterial community, soil metabolome

## Abstract

Organic and microbial fertilizers have potential advantages over inorganic fertilizers in improving soil fertility and crop yield without harmful side-effects. However, the effects of these bio-organic fertilizers on the soil microbiome and metabolome remain largely unknown, especially in the context of bamboo cultivation. In this study, we cultivated *Dendrocalamus farinosus* (*D. farinosus*) plants under five different fertilization conditions: organic fertilizer (OF), *Bacillus amyloliquefaciens* bio-fertilizer (Ba), *Bacillus mucilaginosus* Krassilnikov bio-fertilizer (BmK), organic fertilizer plus *Bacillus amyloliquefaciens* bio-fertilizer (OFBa), and organic fertilizer plus *Bacillus mucilaginosus* Krassilnikov bio-fertilizer (OFBmK). We conducted 16S rRNA sequencing and liquid chromatography/mass spectrometry (LC–MS) to evaluate the soil bacterial composition and soil metabolic activity in the different treatment groups. The results demonstrate that all the fertilization conditions altered the soil bacterial community composition. Moreover, the combination of organic and microbial fertilizers (i.e., in the OFBa and OFBmK groups) significantly affected the relative abundance of soil bacterial species; the largest number of dominant microbial communities were found in the OFBa group, which were strongly correlated with each other. Additionally, non-targeted metabolomics revealed that the levels of soil lipids and lipid-like molecules, and organic acids and their derivatives, were greatly altered under all treatment conditions. The levels of galactitol, guanine, and deoxycytidine were also markedly decreased in the OFBa and OFBmK groups. Moreover, we constructed a regulatory network to delineated the relationships between bamboo phenotype, soil enzymatic activity, soil differential metabolites, and dominant microbial. The network revealed that bio-organic fertilizers promoted bamboo growth by modifying the soil microbiome and metabolome. Accordingly, we concluded that the use of organic fertilizers, microbial fertilizers, or their combination regulated bacterial composition and soil metabolic processes. These findings provide new insights into how *D. farinosus*-bacterial interactions are affected by different fertilization regiments, which are directly applicable to the agricultural cultivation of bamboo.

## 1. Introduction

The soil used in agriculture is largely infertile. It is therefore common agricultural practice to enhance soil fertility by applying large amounts of fertilizer especially nitrogenous fertilizers, to increase crop yield or biomass (Robertson and Vitousek, 2009; Sabir et al., 2021). However, the excessive use of inorganic fertilizers can cause a range of environmental problems such as soil compaction, water eutrophication, energy overconsumption, and air pollution (Toljander et al., 2008; Conley et al., 2009; Guo et al., 2010; Janssens et al., 2010; Divito et al., 2011). Bio-organic fertilizers, are a green technology, which have been documented to improve soil fertility and promote plant growth in the absence of the harmful side-effects of conventional inorganic fertilizers (Bhardwaj et al., 2014; Cui et al., 2018; Maltas et al., 2018; Wu et al., 2018). Therefore, the development and utilization of organic and microbial fertilizers is particularly critical.

Soil microbial communities play an essential role in the regulation of plant growth and development; they account for a large part of the total soil biomass and participate in practically all soil-and plant-associated processes (Fierer et al., 2012). Therefore, the composition and metabolism of the soil microbiome determine the sustainable productivity of agricultural land (van der Heijden et al., 2008; Philippot et al., 2013). Soil microorganisms play an indispensable role in regulating soil fertility, plant yield and quality, stress resistance, and the cycling of carbon, nitrogen, and other nutrients in the ecosystem (Högberg et al., 2001; Pieterse et al., 2016; Bakker et al., 2018; Zou et al., 2022).

Soil microbial communities are extremely sensitive to fertilizers, especially organic and bacterial biofertilizers, as has been demonstrated in a series of previous studies. For example, the application of organic fertilizers such as earthworm manure and mushroom residue enhanced the diversity of the soil bacterial community in degraded grasslands, increased the number of *Actinobacteria* and *Proteobacteria* species, and improved the aboveground biomass of *Leymus chinensis* (Shang et al., 2020). Another study compared the long-term application of chemical and organic fertilizers to the soil of tea plantations. It has been found that organic fertilizers contribute to the uptake of beneficial bacteria within the rhizosphere of tea plants, for example, by significantly increasing the relative abundance of *Burkholderiales* species (Lin et al., 2019). In addition, some studies have found that the partial replacement of chemical fertilizer with organic fertilizer significantly increased the abundance of *Actinobacteria*, and that its influence on bacteria was greater than that on fungi (Ren et al., 2021). The application of microbial fertilizers also alters the composition of the soil microbiome. For instance, the application of microbial fertilizer containing *Bacillus amyloliquefaciens* significantly changed the makeup of the soil bacterial community by increasing the abundance of ammonia oxidizing bacteria (AOB), while not altering the abundance of nitrite oxidizing bacteria (NOB; Xue et al., 2021). Similarly, the addition of *Bacillus-subtilis*-containing microbial fertilizer also increased the abundance of AOB species (Sun et al., 2020). In previous reports, the use of these plant probiotics also improved the health, nutritional composition, and stress resistance of plants (Berendsen et al., 2012; Berlec, 2012; Wei et al., 2015). In addition, the delivery of plant probiotics in bio-organic fertilizers has been particularly effective at improving soil microbial function (Bubici et al., 2019). For instance, the inhibition of banana fusarium wilt using a mixture of organic fertilizer and *B. amyloliquefaciens* W19, was related to its impact on the soil microbial community; specifically, by increasing the number of specific *Pseudomonas* species (Tao et al., 2020). It is worth mentioning that several recent studies have found that the combined effects of nanocompounds and plant growth promotory rhizobacteria are more beneficial for the growth of Fenugreek and maize plants (Kumari et al., 2020; Chaudhary et al., 2021; Agri et al., 2022). However, few studies have focused on the effects of the combined application of organic and microbial fertilizers on the composition of the soil microbiome to improve crop yield, biomass, and nutrient uptake efficiency. Therefore, evaluating how the soil microbial community is altered by the application of organic and microbial fertilizers is crucial for guiding agricultural practice.

Soil metabolites are biomarkers of changes in soil microbial community composition (Swenson et al., 2015). Thus, tracking soil metabolites will help us gain a deeper understanding of how soil microbial communities are affected by different fertilization regimens. Metabolomics has been widely applied to conduct qualitative and quantitative analyzes of changes in the composition of living cells or low-molecular-weight metabolites in the soil, caused by various biological or abiotic factors (Yang et al., 2021). Metabolomics can be combined with microbiomics to identify potential biomarkers of exogenous disturbances in biological systems (Wu et al., 2022). For example, 16S rRNA gene sequencing and gas chromatography–mass spectrometry (GC–MS) metabolomics have been used to study soil microbial communities and the related metabolites (Zhang H. et al., 2020). More recent studies, combining microbiology and non-targeted metabolomics, found that P fertilizer reduced the diversity of the bacterial 16S rRNA and the fungal nuclear ribosomal internal transcribed spacer (ITS) genes, and significantly changed the overall composition of soil bacteria and fungi (Cheng et al., 2022). However, the effects of organic and microbial fertilizers (especially their combined use) on soil metabolites, metabolic pathways, and the structure of soil microbial communities are still largely unknown, especially in bamboo cultivation studies (Rodríguez et al., 2020). Therefore, the combined analysis of the soil microbiome composition, and differences in soil metabolites and the associated metabolic pathways can help us better understand the elusive biological processes occurring in bamboo-growing soils.

*Dendrocalamus farinosus* is a common sympodial bamboo species, which is widely cultivated because of its high yield, strong disease resistance, excellent bamboo fiber characteristics, and fact that it can produce both bamboo shoots and wood (Hu et al., 2011). Previous studies have shown that a high nutrient supply can increase the yield and biomass of bamboo species (Piouceau et al., 2014). However, there have been few studies documenting the use of organic and microbial fertilizers in the cultivation of *D. farinosus*. In addition, it is still unknown how the application of bio-organic fertilizers affects the composition and metabolic characteristics of soil microorganisms to beneficially impact bamboo growth. In the present study, we explored how the application of a bio-organic fertilizer, composed of organic fertilizers, *B. amyloliquefaciens*, and *Bacillus mucilaginosus* Krassilnikov, as well as organic and microbial fertilizer compounds, affected bacterial communities in the soil used for *D. farinosus* cultivation, using 16S rRNA sequencing and liquid chromatography/mass spectrometry (LC–MS). Our main aim was to identify the major microbial groups and soil metabolites that play a role in promoting bamboo growth after soil fertilization.

## 2. Materials and methods

### 2.1. Plants and fertilizers

*Dendrocalamus farinosus* potted plants were grown at the experimental base (location: 31°32 ‘44 “N, 104°41′ 402″ E, altitude 480 m) of the College of Life Science and Engineering, Southwest University of Science and Technology, Mianyang, Sichuan Province. Topsoil (0–20 cm depth) was obtained from the test site and screened to remove rock and plant litter. The soil contained 37% silt, 36% clay, and 27% sand. The soil physical and chemical properties were as follows: pH 6.49 ± 0.01, electrical conductivity 112.93 ± 1.06 μS·cm^−1^, total carbon content: 1220 ± 102 mg kg^−1^, total organic carbon content: 511 ± 12 mg kg^−1^, total nitrogen content: 353 ± 11 mg kg^−1^, total phosphorus content: 180 ± 9 mg kg^−1^, available nitrogen content: 142 ± 11 mg kg^−1^, available phosphorus content: 22.4 ± 7.3 mg kg^−1^.

Three fertilizers were used in this study. The organic fertilizer (organic matter ≥45%, N + P_2_O_5_ + K_2_O ≥ 5%, N [1.4%], P [4.5%], K [1.7%]) was purchased from Henan Lotus Environmental Technology Fertilizer Corporation (Henan, China). The *B. mucilaginosus* microbial fertilizer (microbial agent, 10 billion colony forming units [CFU]/g) was purchased from Huanwei Biology; *B. mucilaginosus* decomposes K, Si, and P from soil minerals such as feldspar, mica, and apatite, thus increasing K and P supply to the soil and ultimately crop yields (Liu et al., 2006). The *B. amyloliquefaciens* microbial fertilizer (microbial agent, 100 billion CFU/g) was purchased from Nongbao Biology; *B. amyloliquefaciens* is a typical plant-growth-promoting rhizobacterium (PGPR), which promotes plant growth and improves nitrogen use efficiency (Xue et al., 2021).

### 2.2. Sample collection and preparation

First, each type of fertilizer was added to the collected topsoil. 50 kg of the soil-fertilizer mixture was then used to fill each plastic pot top diameter: 44 cm, bottom diameter: 28 cm, height: 30 cm. The amount of organic manure used for each plant was about 500 g (1–2 g of *B. mucilaginosus* per kg of pot soil and 0.4–0.8 g of *B. amyloliquefaciens* per kg of pot soil). The following six treatment conditions were used: (1) Control: no fertilization; (2) organic fertilizer (OF): 500 g of organic fertilizer; (3) *B. mucilaginosus* (BmK): 75 g *B. mucilaginosus*; (4) *B. amyloliquefaciens* (Ba): 30 g *B. amyloliquefaciens*; (5) OFBmK: 500 g of organic fertilizer and 75 g of *B. mucilaginosus*; and (6) OFBa: 500 g of organic fertilizer and 30 g of *B. amyloliquefaciens*. Three biological replicates were performed for each treatment condition. Healthy potted bamboo plants of similar size were transplanted into pots containing the different soils and watered. The plants were then watered weekly during the growing period, by letting the water run through the potting soil. *D. farinosus* used in the experiments are all annual bamboos. After the *D. farinosus* plants had produced new shoots, five soil cores (2 cm topsoil removed, 10 cm depth, 2.5 cm diameter) were extracted from each pot with a soil sampling probe, using the five-point sampling method (Ni et al., 2008). The corresponding soil samples were then mixed and divided into two groups. One group of samples was stored at 4°C for the determination of soil physical and chemical properties and the analysis of soil enzyme activities, and the other group was stored at −80°C for the analysis of the soil microbiome and metabolome.

### 2.3. Analysis of bamboo phenotypes

Ninety days after transplantation of bamboo plants, photographs were taken to document the above-ground growth of *D. farinosus*. The fresh weight, length, outer diameter, and wall thickness of each bamboo internode were determined. Leaf photosynthetic pigments were extracted with ethanol-acetone solution and quantified spectrophotometrically (Zhang Y. et al., 2020). Leaf nitrogen content was determined using a Chlorophyll Analyzer (TYS-4 N model, Beijing Zhongke Weihe Technology Development Co., Ltd. China). Soluble sugar concentration within the leaves was measured using the anthrone colorimetric method (Mo et al., 2020). The total number of new shoots was counted at the end of the *D. farinosus* growing period. The photosynthetic rates of the leaves were measured using a photosynthesis measurement system (LCPRO-SD, ADC Bioscitical, Inc., United Kingdom).

### 2.4. Determination of soil enzyme activity

Plant residue was removed from fresh soil by sieving through a 100 mesh sieve. Soil sucrase activity was determined spectrophotometrically using 3,5-dinitrosalicylic acid, and was defined as the unit of enzymatic activity (U/g) that produces 1 mg of reducing sugar per g of soil per day. Soil urease activity was determined using the indophenol blue spectrophotometric method, and was defined as the unit of enzymatic activity (U/g) for the production of 1 mg ammoniacal nitrogen (NH3-N) per day in the soil. Soil acid phosphatase activity was measured using a benzene-para-phosphorus spectrophotometric method, and was defined as units of enzyme activity (U/g) based on the release of 1 nmol of phenol per g of soil per day. Soil peroxidase activity was determined spectrophotometrically using pyrogenic gallic acid and was defined as the unit of enzyme activity (U/g) in soil producing 1 mg of gallic acid per day. Soil dehydrogenase activity was determined spectrophotometrically using 2,3,5-triphenyltetrazolium (TTC), with one unit of enzyme activity (U/g) as an increase in optical density (OD) of 0.01 per g of soil per mL of reaction per h (Fu et al., 2022). The enzymatic activity kit (Beijing Solabao Technology Co, LTD) was used to assay the soil enzymatic activity in three biological replicates.

### 2.5. DNA extraction and library construction for 16S rRNA amplicon sequencing

Soil genomic DNA was extracted using a DNA extraction kit (DNeasy PowerSoil Kit, QIAGEN). DNA concentration was measured using agarose gel electrophoresis and NanoDrop2000 (Thermo Fisher Scientific, Waltham, MA, United States). The bacterial 16S rRNA gene V3–V4 high variant region was amplified in a 30 μl reaction volume with the universal primers 343F: 5’-TACGGRAGGCAGCAG-3′ and 798R: 5’-AGGGTATCTAATCCT-3′ (Nossa et al., 2010). The amplification reaction comprised 15 μl 2 × GFlex PCR Buffer, 1 μl primer R (5 pmol/μL), 1 μl primer F (5 pmol/μL), 1 μl (50 ng) template, 0.6 μl TKS GFlex DNA polymerase (1.25 U/μL), and 11.4 μl ddH_2_O (Takara City, Japan). The amplification program was as follows: 94°C for 5 min, 94°C for 30 s, 56°C for 30 s, 72°C for 20 s, 26 cycles at 72°C for 5 min, and 4°C hold. The PCR products were detected using electrophoresis and then purified using magnetic beads. The purified products were used as templates in a second round of PCR amplification, and again detected using electrophoresis and purified using magnetic beads. After purification, the PCR products were quantified by Qubit and equal amounts of purified amplicons were mixed for subsequent sequencing (Wu et al., 2015). The samples were assayed on an Illumina NovaSeq6000 platform (Illumina Inc., San Diego, CA; OE Biotech Company; Shanghai, China) with two paired end read cycles and 250 bases per cycle. All treatment groups were assayed in triplicate.

### 2.6. Processing and analysis of 16S rRNA sequencing data

Paired end reads were preprocessed using TRIMMIC (version 0.35) to detect and remove ambiguous bases (Bolger et al., 2014). FLASH (version 1.2.11) was then used to assemble the sequences (Magoč and Salzberg, 2011). To obtain clean tag sequences, sequences containing ambiguous bases and sequences with a single base repeat 8–200 bp in length were removed from the paired end sequences using QIIME (version 1.8.0) software (Caporaso et al., 2010). Finally, chimeric reads were removed using vSearch software (Rognes et al., 2016). Sequence similarity was determined by classifying sequences into operational taxonomic units (OTUs); sequences with more than 97% similarity were grouped into the same OTU. OTUs were annotated and bioinformatically analyzed using QIIME, Greengenes, and RDP classifier software (Nossa et al., 2010). Standard bioinformatics analysis was performed by Shanghai OE Biotechnology Co., Ltd., China. Principal component analysis (PCA) was used to analyze the differences in the composition of multiple data sets. Histograms were used to indicate the order of the top 15 most abundant species at different taxonomic levels. 16S rRNA sequencing data were annotated using the Greengenes database and the PICRUSt software package [16S Clusters of Orthologous Genes (COG) and Kyoto Encyclopedia of Genes and Genomes (KEGG) function prediction] to predict known microbial gene functions (Langille et al., 2013).

### 2.7. Liquid chromatography/mass spectrometry non-targeted metabolomics

1 g of soil sample was mixed with 10 μl of L-2-chlorophenylalanine (0.3 mg/ml) and 1 ml of methanol–water (v/v = 1:1) solution, with methanol as the solvent, and placed at −20°C for 2 min. The soil samples were then crushed with a grinder for 2 min, transferred into a centrifuge tube, and centrifuged for 10 min (7,700 rpm, 4°C). 2.5 ml of the supernatant was freeze-dried in a clean centrifuge tube, then resuspended in 400 μl of methanol–water (v/v = 1:4), shaken for 1 min and centrifuged for 10 min (12,000 rpm, 4°C). The final reaction volume was 150 μl and the LC–MS analysis conditions were as follows: ACQUITY UPLC HSS T3 column (100 mm × 2.1 mm, 1.8 μm), column temperature 45°C, mobile phase A, water (containing 0.1% formic acid), mobile phase B, acetonitrile (containing 0.1% formic acid), flow rate 0.35 ml/min, injection volume 2 μl. Electrospray ionization (ESI) was used as the ion source, and the mass spectral signals were acquired by positive and negative ion scanning (Lai et al., 2020).

Progenesis QI v2.3 was used to preprocess the MS data (Zhang et al., 2016). The substances were quantified using the Human Metabolome Database (HMDB; Wishart et al., 2018), Lipidmaps (Yang et al., 2021), and METLIN (Xue et al., 2020). The self-built MS library from Shanghai Lu-Ming Biotech Co., Ltd., Shanghai, China was used to generate the metabolomic data matrix table. The metabolite intensities of 18 sets of samples and quality control (QC) samples were displayed on a Box plot; all mass spectral intensities were presented as log10 values. Bar graphs were used to present the number of accurately characterized positive and negative substance peaks and metabolites (Lai et al., 2020).

### 2.8. Differential metabolite screening and metabolic pathway analysis

The orthogonal partial least squares discriminant analysis (OPLS-DA) method was used to screen DEMs. The corresponding OPLS-DA model was established to obtain the R^2^ and Q^2^ values of the stochastic model to obtain the metabolite variable important in projection (VIP) values. Student’s t-test and fold change analyzes were used to assess differences in metabolites between two groups (Zhao et al., 2020). In this study, the thresholds for screening DEMs were VIP values were > 1 and *p* < 0.05, respectively. Based on the screening results, the number of up-and down-regulated DEMs in each treatment group was counted, and the DEM expression patterns of the top 10 ranked VIP values were presented. To analyze intergroup DEM differences, Venn diagrams were used to evaluate the distribution of specifically-expressed or co-expressed DEMs between treatment groups. The soil metabolite data matrix was compared against KEGG^1^ datasets to determine the KEGG IDs of the metabolites; the metabolic pathways associated with the DEM were filtered according to the annotated results. The screening threshold for metabolic pathway enrichment analysis of DEMs was *p* < 0.05 (Zhang Y. et al., 2020). Based on the metabolite data matrix, galactose, nucleotide, and amino acid metabolism under different fertilization conditions were then evaluated. Heat maps were used to present the expression patterns of different metabolites under various test conditions (Horemans et al., 2015).

### 2.9. Statistical analysis

GraphPad Prism 8.0.1 software (Microsoft Window, United States) software was used to generate bar graphs and box plots. Statistical analysis was performed using SPSS Statistics 22.0 software (IBM, United States). Statistical plots (e.g., heat maps, principal component analysis (PCA), Venn diagram) used in this study were drawn using the OmicShare online analysis platform^2^. Image layout was performed using Adobe Illustrator CS6 (Adobe, United States).

## 3. Results

### 3.1. Organic and microbial fertilization promotes *Dendrocalamus farinosus* growth

**Figure 1:**
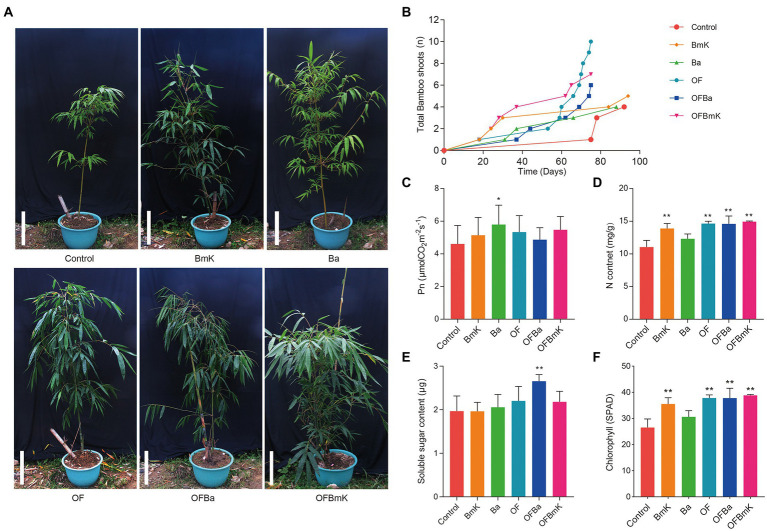
Effect of bio-organic fertilization on the growth of *Dendrocalamus farinosus*. **(A)** The phenotypes of *D. farinosus* 90 days after the initiation of fertilization. Scale bar, 50 cm. **(B)** Statistical analysis of the total number of bamboo shoots produced by each plant and the duration of bamboo shoot formation. Determination and analysis of net photosynthetic rate **(C)**, nitrogen content **(D)**, soluble sugar content **(E)**, and chlorophyll content **(F)** in bamboo leaves. One-way ANOVA and Tukey post-hoc tests were used to calculate the differences between treatment groups. *p < 0.05; **p < 0.01. BmK–OFBmK refers to the different fertilization regimens: BmK, *Bacillus mucilaginosus Krassilnikov*; Ba, *Bacillus amyloliquefaciens*; OF, organic fertilizer; OFBa, bio-organic fertilizer (containing *Bacillus amyloliquefaciens*); OFBmK, bio-organic fertilizer (containing *Bacillus mucilaginosus Krassilnikov*).

To investigate the impact of fertilization on the growth of *D. farinosus* we measured the above-ground growth phenotypes of bamboo shoots under six fertilization conditions (Figure 1). The plants in all fertilized groups grew better (i.e., taller plants, more and greener leaves) than those of the control group and had more bamboo shoots, which also appeared earlier (Figure 1A). The total shoot production of the Ba group was comparable to that of the control group. Meanwhile, all the other test groups produced more shoots than the control group (Figure 1B). Moreover, shoot production was significantly higher in the combined organic and bacterial fertilizer treatment groups than in the other treatments. The earliest day of shoot emergence in the BmK and OFBmK groups was earlier than that in the control group (on the days 18 and 37, respectively) (Figure 1B). The net photosynthetic rate in the leaves of the Ba group was significantly higher than that of the control group (Figure 1C). Leaf nitrogen content of the BmK, OF, OFBa, and OFBmK groups was significantly higher than that of the control group (Figure 1D). The soluble sugar content in the leaves of the OFBa group was significantly higher than that of the control group (Figure 1E). In addition, the relative chlorophyll content of the BmK, OF, OFBa, and OFBmK plants was significantly higher than that of the control group (Figure 1F). Therefore, we further analyzed the internode phenotypes, and the lignin and cellulose content of the bamboo plants treated with organic and bacterial fertilizers. We found that the phenotypes of plants in the compound fertilizer treatment groups were significantly better than those in the control group (Supplementary Figure S1). Collectively, these findings suggest that the soil composition and fertility changed dramatically after combined organic and bacterial fertilization.

### 3.2. The application of organic and microbial fertilizers alters the soil enzyme activity and enhances soil fertility

**Figure 2:**
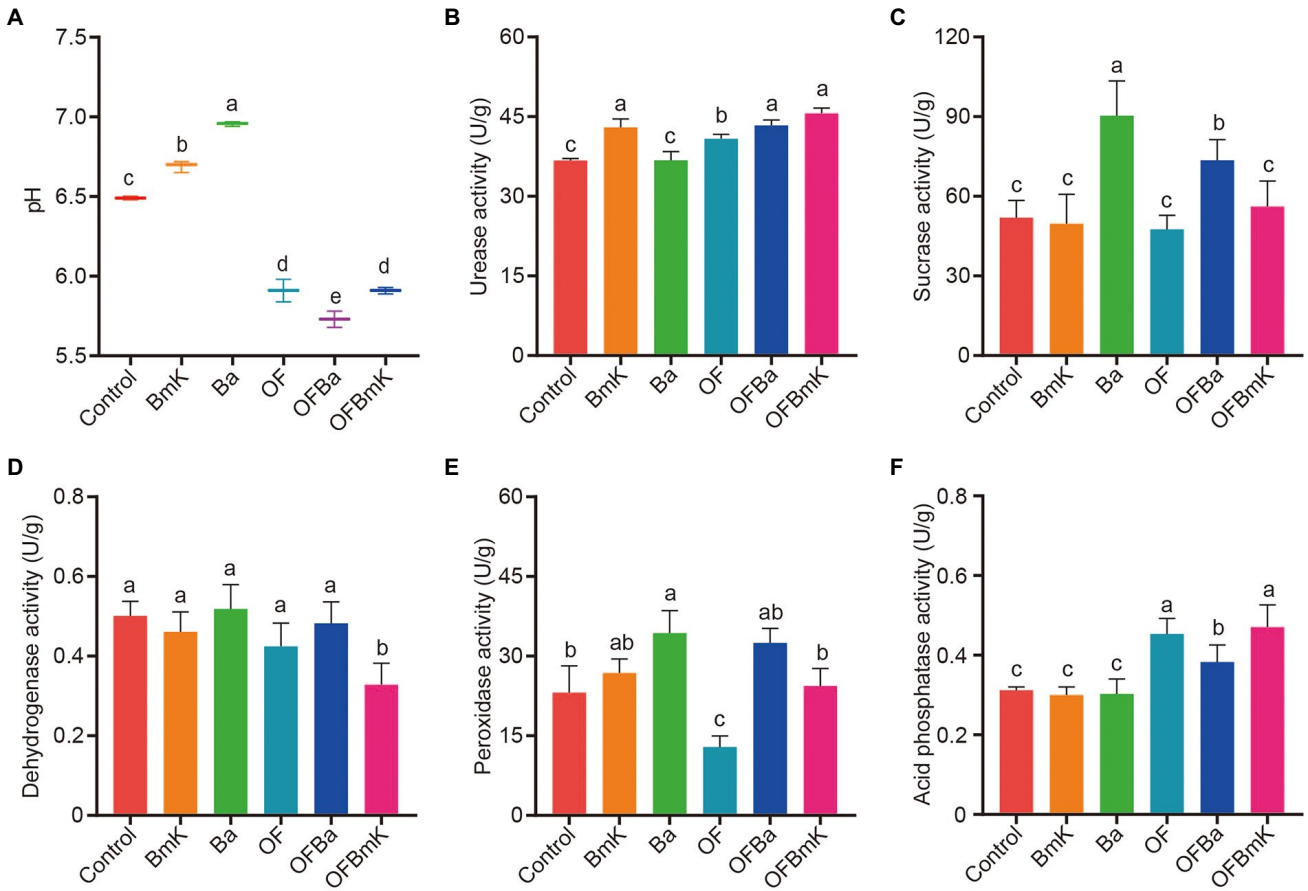
Effects of bio-organic fertilization on the pH and enzymatic activity in the soil. Soil pH **(A)**, urease activity **(B)**, sucrase activity **(C)**, dehydrogenase activity **(D)**, peroxidase activity **(E)**, and acid phosphatase activity **(F)** 90 days after the initiation of *Dendrocalamus farinosus* fertilization. One-way ANOVA and Tukey post-hoc test were used to calculate the differences between treatment groups; different letters represent significant differences between the mean values (*p* < 0.05, *n* = 3).

The levels of carbon, nitrogen, and phosphorus and the activities of soil enzymes are critical indicators of soil fertility. Therefore, we next explored changes in soil properties and the activities of important soil enzymes after fertilization. Firstly, we measured the pH, total carbon (TC), total organic carbon (TOC), total nitrogen (TN), total phosphorus (TP), and available nitrogen (AN) in the six test soils types. We found that soil treatment with OF, OFBa, or OFBmK significantly reduced soil pH (from 6.49 to 5.91, 5.73 and 5.90, respectively; Figure 2A), which made the soil more suitable for bamboo growth. The experimental results also showed a significant increase in the amounts of TC, TOC, TN, TP, and AN in the soil after fertilization with OF, OFBa, and OFBmK (Supplementary Table S1). We further measured the activity of urease, sucrase, dehydrogenase, peroxidase, and acid phosphatase in the six test soils (Figures 2B–F). We found that sucrase activity positively correlated with soil fertility. The catalytic product of urease is ammonia, which can be absorbed and utilized by plants. The level of urease activity can be indicative of the nitrogen utilization efficiency of plants. In addition, acid phosphatase activity is positively correlated with soil carbon and nitrogen content, and is a reliable indicator of the strength of phosphorus conversion in the soil. We found that the urease activities of soil treated with BmK, OF, OFBa, or OFBmK increased by 17.1, 11.2, 18.1%, or 24.3%, respectively, compared with the control group (Figure 2B). The sucrase activity of soil treated with Ba or OFBa (74.1% or 41.7%, respectively) was significantly higher than that of the control group; no significant changes were detected in the other groups (Figure 2C). The acid phosphatase activities of the OF, OFBa, or OFBmK treatment groups were significantly increased by 45.2, 22.6%, or 50.9%, respectively (Figure 2F). These results indicate that the application of organic and microbial fertilizers promotes bamboo growth by altering the physical and chemical properties of the soil and enhancing the activity of soil enzymes.

### 3.3. Bio-organic fertilization significantly alters soil microbiome composition

**Figure 3:**
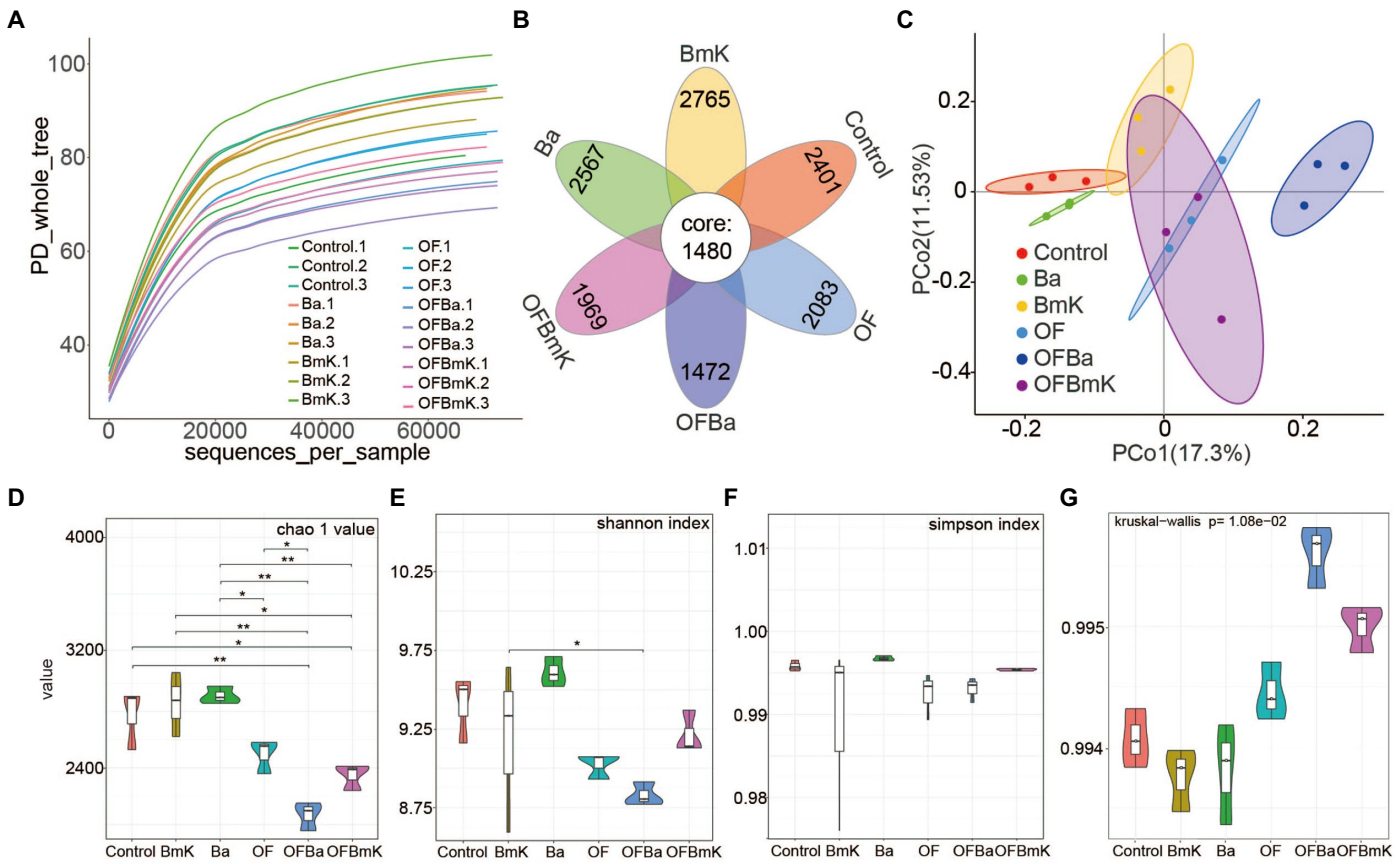
Microbial diversity in the rhizosphere of *Dendrocalamus farinosus* under various fertilization conditions. **(A)** Diversity index dilution curve, where each curve represents a sample, the abscissa is the depth of random sampling, and the ordinate is the exponential value. **(B)** Venn diagram of the operational taxonomic units (OTUs) shared by all six conditions, and the number on each petal represents the total OTUs of each sample minus the number of shared OTUs. **(C)** Principal component analysis based on the binary_jaccard distance matrix. Analysis of Chao1 index **(D)**, Shannon index **(E)**, and Simpson index **(F)** related to bacterial alpha diversity. The Kruskal–Wallis algorithm was used to calculate the significance of the differences between treatment conditions (*p < 0.05, **p < 0.01). **(G)** The Goods_coverage index was used to evaluate whether the sequencing results could reliably represent the real composition of microorganisms in the samples.

To explore the changes in the composition of the soil microbial population after fertilization, we performed 16S rRNA sequencing on 18 soil samples (three biological replicates for each of the six treatment groups). The number of valid tags in the sequencing data ranges from 67,779 to 74,623 (Supplementary Figure S2). When we increased the number of sampled sequences to 60,000, the diversity index dilution curves for different samples began to flatten (Figure 3A), indicating that the amount of sequencing data per sample was reasonable and could accurately reflect the diversity of microorganisms in the sample. We detected 3,881 (Control), 4,236 (BmK), 4,047(Ba), 3,536 (OF), 3,449 (OFBmK), and 2,952 (OFBa) OTUs in the soil samples. Of these, 1,480 OTUs were shared between the treatment groups (Figure 3B; Supplementary Table S2). The PCA results showed that the differences within each treatment group were small. Significant changes in OTUs were observed in the OF, OFBa, and OFBmK groups compared to the control group, and non-significant subtle changes were observed in the Ba and BmK groups compared to the control groups (Figure 3C). The results of the α-diversity analysis showed that the Chao1 values of the OFBa and OFBmK groups were significantly lower than that of the control soil; however, there was no significant difference between the Ba, BmK, and OF groups (Figure 3D). The Shannon index of the soil microbial community after fertilization showed a downward trend compared to the control group, with the OFBa group showing significantly lower values than the Ba group (Figure 3E). We further evaluated the microbial coverage of each sample and found that the Simpson indexes and Goods_coverage values for all samples were close to 1 (Figures 3F,G), indicating that the species richness and evenness of each sample reached the highest level. Together, these results suggest that the microbial richness of the soil was significantly reduced after the combined application of organic and microbial fertilizers, and especially after the OFBa treatment.

### 3.4. Bio-organic fertilization significantly changes the metabolic processes of the dominant soil bacteria

**Figure 4:**
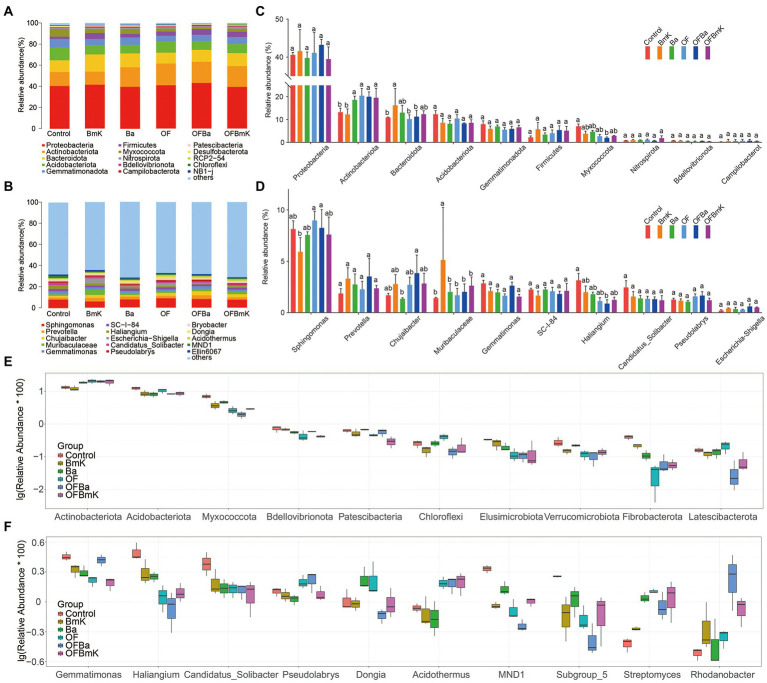
Effect of different fertilization treatments on the soil microbial community composition at the phylum and genus levels. Phylum **(A)** and genus **(B)** levels of the top 15 most abundant microorganisms. The species belonging to the top 10 most abundant phyla **(C)** and genera **(D)** were selected, and the differences between treatment groups were calculated using the one-way ANOVA and Tukey post-hoc test; different letters represent significant differences between the mean values (*p* < 0.05, *n* = 3). Boxplot of differential species at the level of phylum **(E)** and genus **(F)** levels; the top 10 diverse species were selected and analyzed by ANOVA to compare the abundance of dominant species within and between treatment groups.

To understand how the composition and abundance of microorganisms in the soil changed under different treatment conditions, we characterized the top 15 most abundant bacterial species according to phylum, class, order, family, and genus in different treatment groups (Figures 4A–D; Supplementary Figure S3). We found that the different treatment conditions did not change the presence of the dominant microbes at the phylum level. The following phyla were most abundant: *Proteobacteria*, *Actinobacteriota*, *Bacteroidota*, *Acidobacteriota*, *Gemmatimonadota*, *Firmicutes*, and *Myxococcota*; among these, only the relative abundance of *Actinobacteriota*, *Bacteroidota*, and *Myxococcota* were altered (Figures 4A,C). However, the impact of fertilization on the soil microbial communities at the genus level were more complex. We found that both the composition and relative abundance of dominant microorganisms at the genus level in the soil changed significantly with the addition of bio-organic fertilizers (Figures 4B,D).

**Figure 5:**
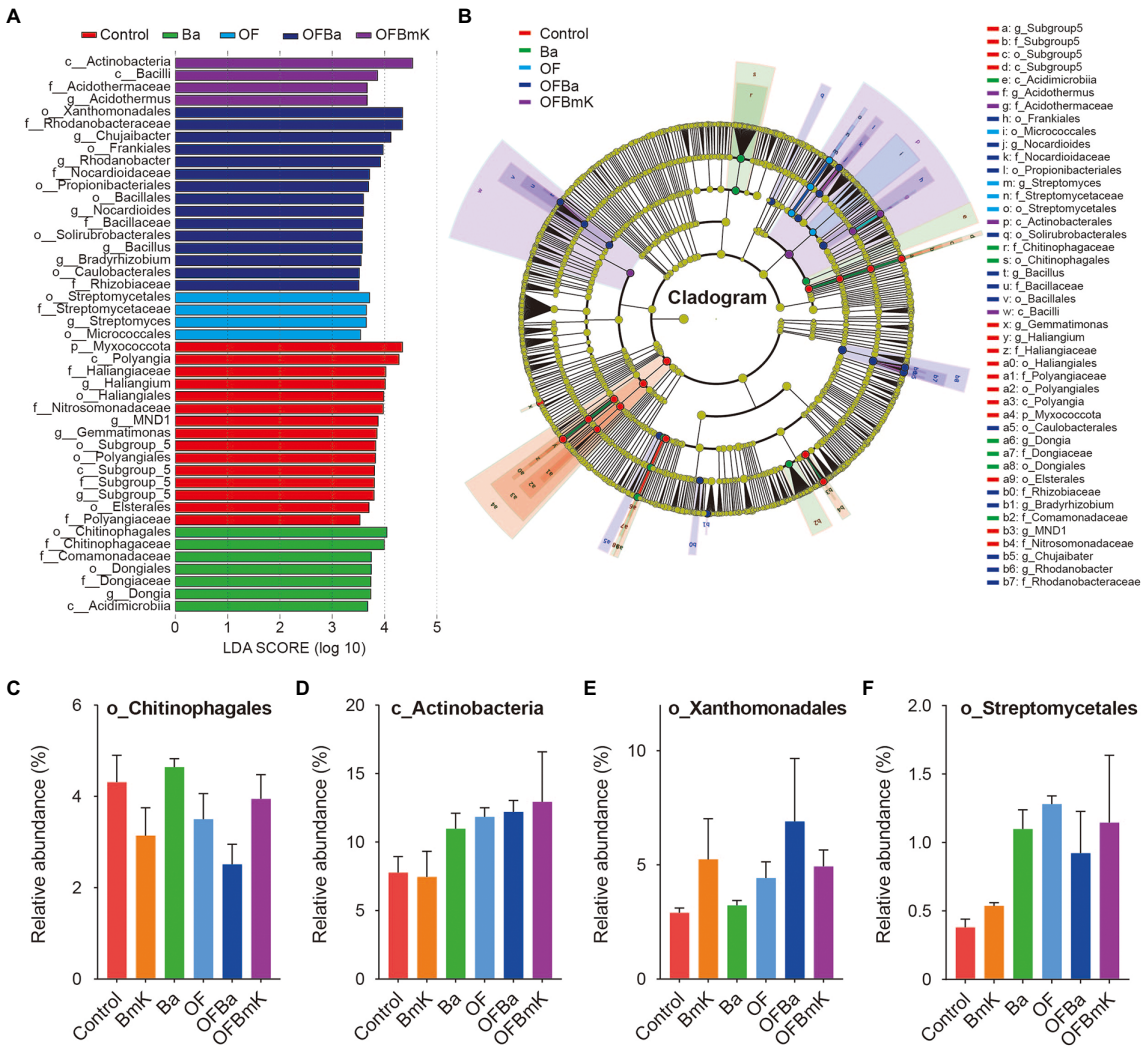
Analysis of different microbial species in the soil under different fertilization conditions. **(A)** Biomarkers associated with the microorganisms present in the soil under different fertilization conditions. Different colored bars indicate species with relatively high abundances in different groups (LDA Score > 3.5). **(B)** Annotated branching diagram of different microbial species. The yellow nodes represent species that do not differ significantly between treatment conditions. Each section represents a phylum, class, order, family, and genus from the inside out. Relative abundances of the single most abundant biomarkers in the Ba **(C)**, OFBmk **(D)**, OFBa **(E)**, and OF **(F)** groups.

We further compared the top 10 differential microbial species at different taxonomic levels among the treatment groups (Figures 4E,F; Supplementary Figure S4). In the analysis of the top 10 differential phyla, the ANOVA algorithm was used to show that all the treatments except BmK increased the relative abundance of bacteria belonging to the *Actinobacteriota* phylum. In contrast, all treatments (especially OFBa and OFBmK) reduced the relative abundance of the *Acidobateriota*, *Myxococcota*, and *Bdellovibrionota* phyla (Figure 4E). In the analysis of the top 10 differential genera, all fertilization treatments decreased the relative abundance of *Gemmatimonas*, *Haliangium*, and *Candidatus Solibacter*, while increased the relative abundance of *Streptomyces* and *Rhodanobater* (Figure 4F). To obtain a representative snapshot of soil microorganism composition after fertilization, we used linear discriminant analysis (LDA) coupled with effect size measurements (the LEfSe analysis method) to analyze the contribution of different bacterial species (LDA SCORE > 3.5). The results showed that in the OFBa treatment group the number of different biomarkers was significantly higher than that in the other fertilization groups (Figures 5A,B). Seven biomarkers such as *o_Chitinophagales* were present in the Ba group (Figures 5A,C). OFBmK-treated soil contained four types of biomarkers, such as *c_Actinobacteria* (Figures 5A,D). The OFBa group contained 15 biomarkers, such as *o_Xanthomonadales* (Figures 5A,E), and the OF group contained four types of biomarkers, such as *o_Streptomycetales* (Figures 5A,F).

In addition, we predicted the functions of the identified microbial by performing COG and KEGG analyzes of the 16S rRNA sequences (Supplementary Figure S5; Supplementary Table S3). The results showed that the metabolic pathway changes following soil treatment with OF, OFBa, or OFBmK were clustered in one group, and were significantly different from those of the Ba, BmK, and control groups. This also implies that the use of organic and microbial fertilizers (especially in the OFBa treatment) significantly affects the metabolic processes of the dominant microorganisms.

### 3.5. The soil primary metabolic network changes significantly after the application of bio-organic fertilizers

**Figure 6:**
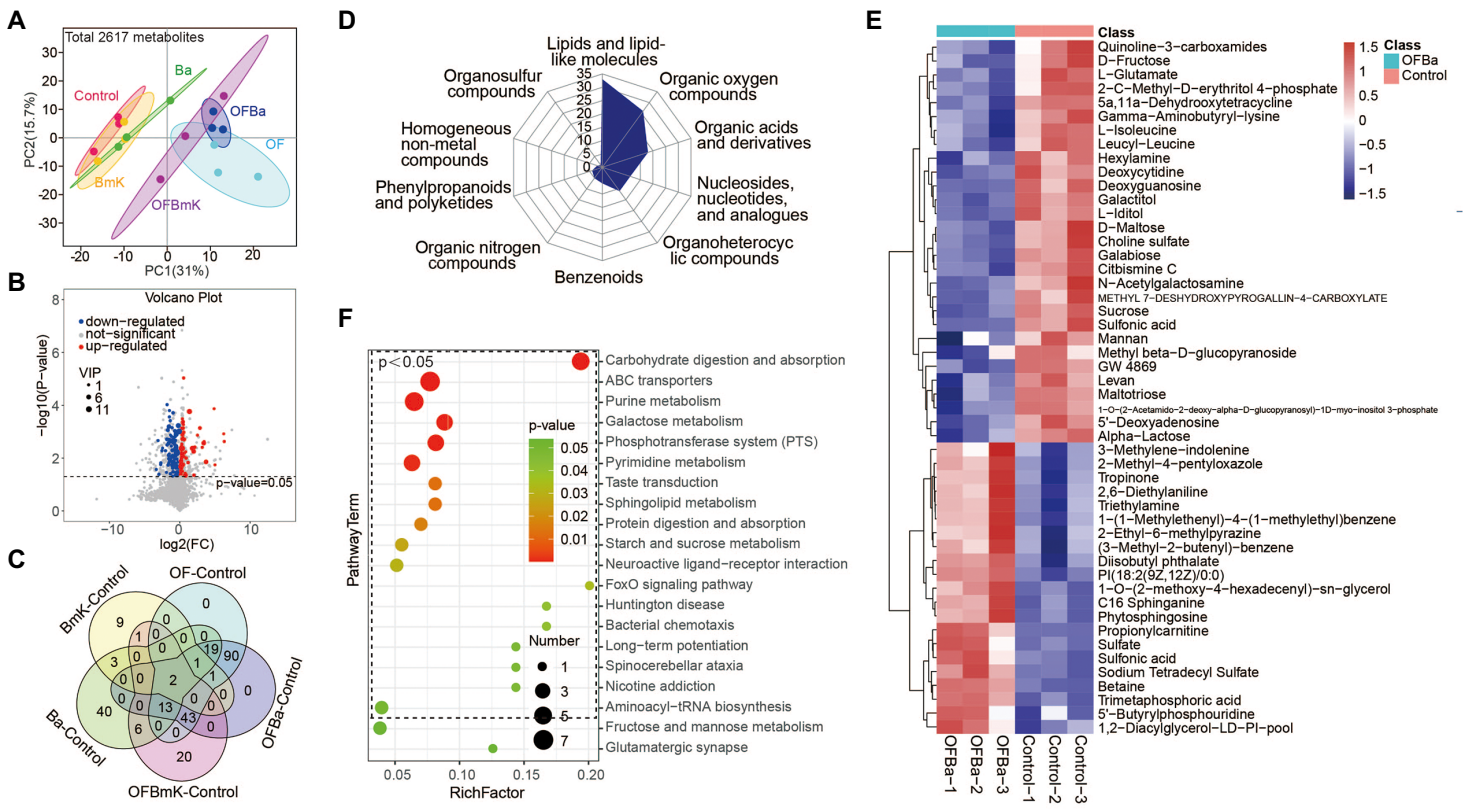
Analysis of soil metabolites in the OFBa fertilization group. **(A)** Principal component analysis of soil metabolites in the different fertilization treatment groups. **(B)** Volcano plot of soil microbial metabolites in the OFBa treatment group. **(C)** Intersections of differential metabolites (DEMs) between fertilization and control groups. Classification of soil DEMs in the OFBa treatment group **(D)** and DEM content change analysis (top 50) **(E)**. **(F)** Enrichment analysis of DEM-associated metabolism pathways (top 20) in the OFBa treatment group.

To further understand the changes in the soil metabolic profile after fertilization, we performed LC–MS non-targeted metabolomics analysis on six test soils. We identified a total of 2,617 metabolites. The PCA results showed that the aggregation locations of the different treatment groups were significantly different, indicating that the soil metabolic profiles changed significantly with each type of fertilization (Figure 6A). Based on the OPLS-DA model analysis (VIP > 1, *p* < 0.05; Figure 6B; Supplementary Figures S6A–D), we found that the BmK group had 17 DEMs, the Ba group had 84 DEMs, the OF group had 112 DEMs, the OFBa group had 169 DEMs, and the OFBmK group had 85 DEMs, compared with the control group (Supplementary Table S4). The OFBa group had the highest number of DEMs, which further suggests that the combined fertilization with OF and Ba caused the greatest changes in soil microorganism composition and their metabolic profiles. In addition, we found that the metabolite levels of galactitol and 1-O-(2-acetamido-2-deoxy-alpha-D-glucopyranosyl)-1D-myo-inositol 3-phosphate underwent the changes in all compared groups (Figure 6C). These results suggest that changes in their contents are sensitive to fertilization and could serve as biomarkers of changes in soil metabolism.

We then classified and counted DEMs (Figure 6D,E; Supplementary Figures S6, S7) among the treatment groups. We found that the top 3 DEM enrichment groups (produced under all fertilization conditions) were dominated by lipids and lipid-like molecules, organic oxygen compounds, and organic acids and derivatives, respectively (Figure 6E; Supplementary Figures 6E–H). We also found that the OFBa group had a higher number of the first two types of metabolites than the other groups. We next investigated which key metabolic pathways were affected by changes in metabolites after fertilization, and whether these differences were the result of metabolic changes. We used the KEGG database to conduct the metabolic pathway enrichment analysis. The results showed that practically all the top 5 enriched pathways, involving galactose metabolism, were present in the fertilization groups, compared with the control group. Moreover, the phosphotransferase system (PTS) and purine metabolism were significantly enriched following the application of organic and biological fertilizers, and especially after their combined use (Figure 6F; Supplementary Figure S8; Supplementary Table S5). In addition, metabolic pathways of carbohydrate digestion and absorption were significantly enriched in the OFBa group (Figure 6F).

**Figure 7:**
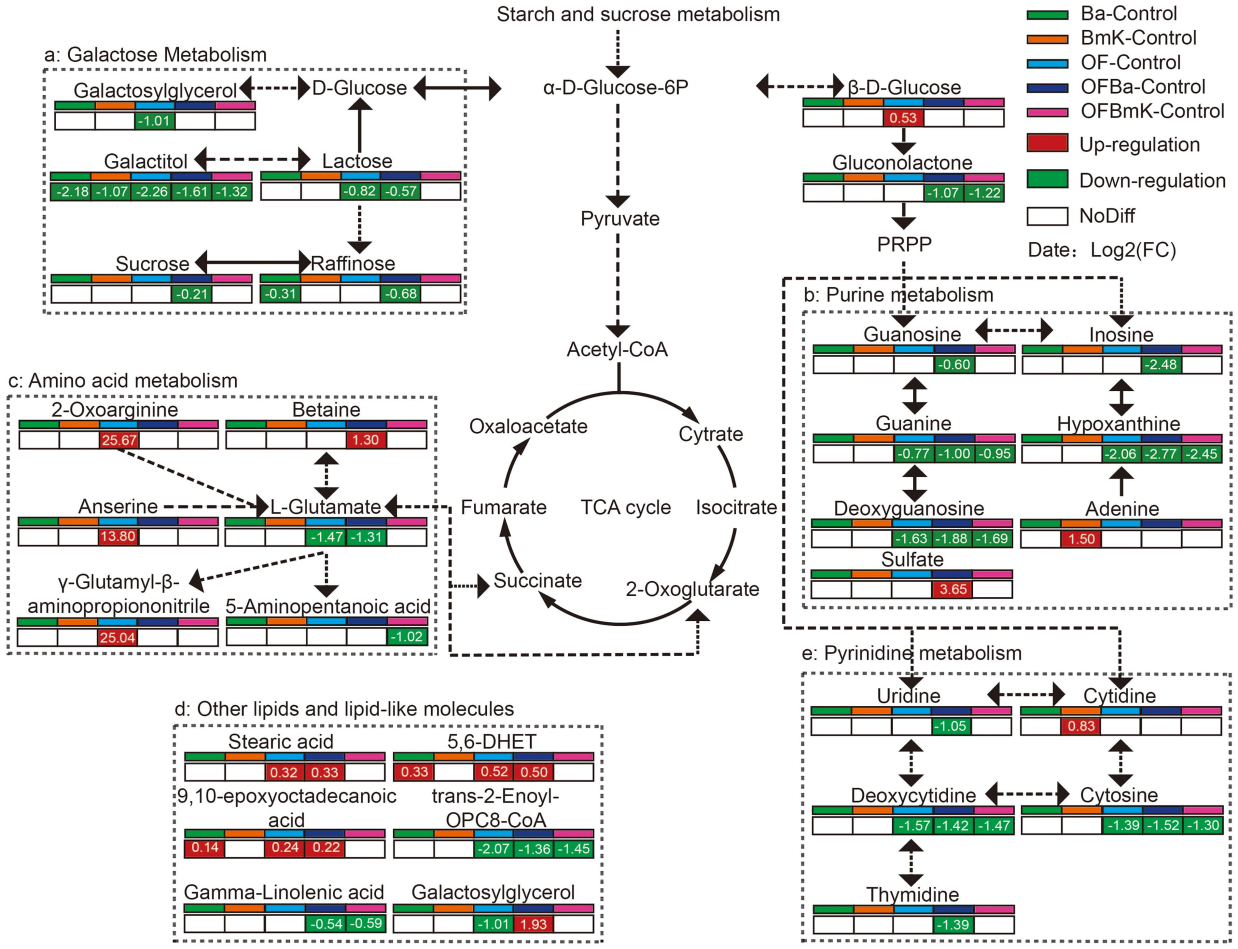
The soil primary metabolic network of the carbohydrate, amino acid, purine, and pyrimidine metabolic pathways after fertilization.

It is worth mentioning that carbohydrate, amino acid, purine-pyrimidine, and other metabolic pathways belong to the soil primary metabolic network. Therefore, we subsequently compared the content of each metabolite in the soil primary metabolic network and found that the key metabolites in this network changed significantly after fertilization (Figure 7). The levels of metabolites in the network (e.g., galactitol, glutamate, and OPC8-CoA) were mostly decreased: galactitol (−2.26 to −1.07), guanine (−1.00 to −0.77), cytosine (−1.52 to −1.30), and deoxycytidine (−1.57 to −1.42). Analysis of the galactose metabolic pathway showed that galactitol levels were significantly decreased by the different types of fertilization (−2.26 to −1.07). Moreover, the levels of lactose, sucrose, and raffinose in the OFBa group were decreased by 0.57-, 0.21-, or 0.68-fold, respectively, than those in the control group. Metabolites such as guanine, hypoxanthine, deoxycytidine, deoxyguanosine, and cytosine in the purine and pyrimidine metabolic pathways were also significantly lower in soil samples from the OF, OFBa, and OFBmK groups, compared to the control. In addition, amino acid metabolic pathways, especially 2-oxoarginine (25.87-fold), anserine (13.80-fold), and γ-glutamyl-β-aminopropiononitrile (25.04-fold) were significantly activated in the OF group, compared to the control group. Furthermore, we found that betaine levels were significantly higher in OFBa group (1.30-fold), while L-glutamate levels were significantly lower (1.31-fold) than those of the control group. Taken together, these results suggest that the application of organic and biological fertilizers, particularly OFBa, regulates bamboo growth and development by altering the soil microbiome composition, which in turn alters the soil primary metabolic network.

### 3.6. Correlation regulatory network of bamboo phenotype, soil fertility, soil DEMs, and dominant soil microbial

**Figure 8:**
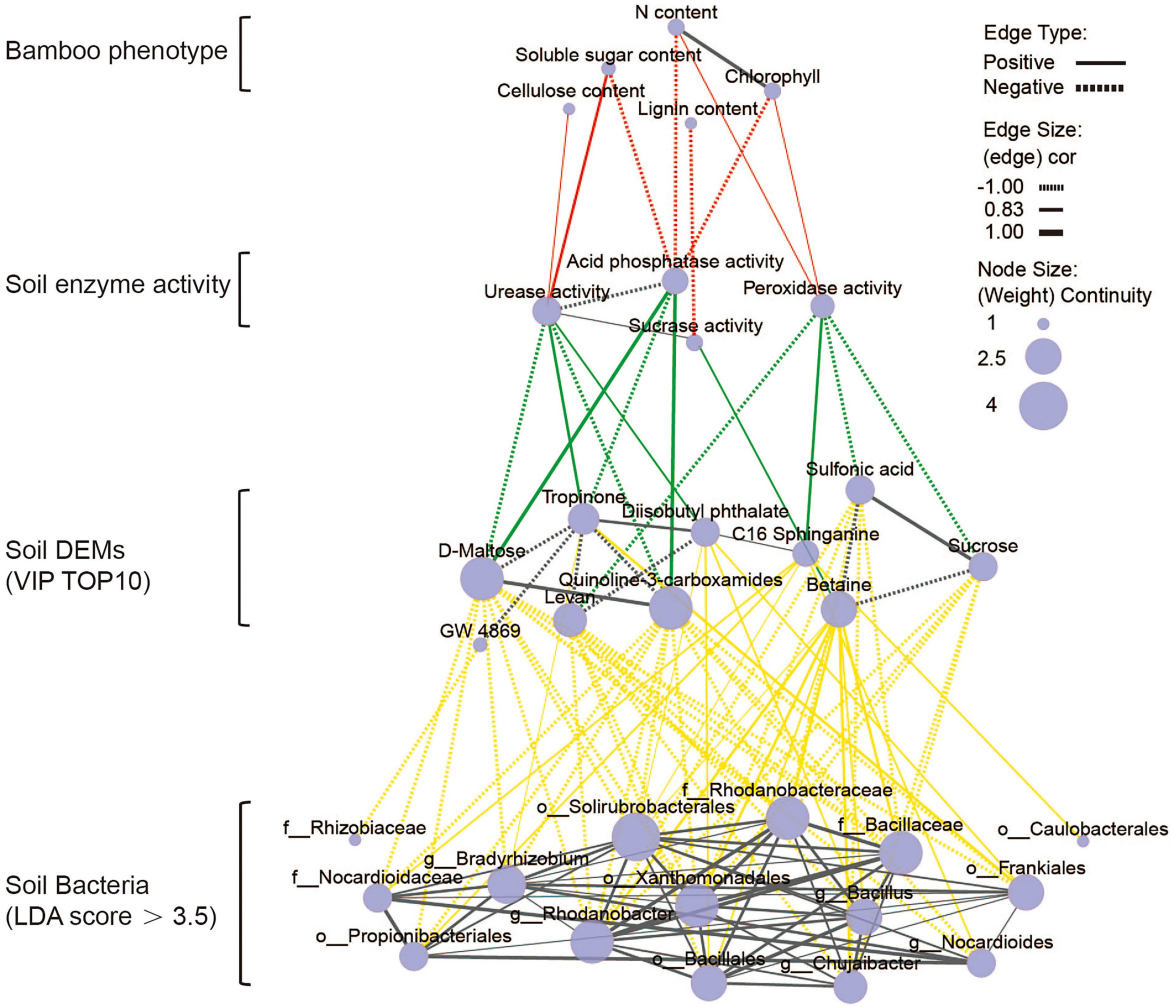
Correlation network between the *Dendrocalamus farinosus* growth phenotype and soil enzyme activity, soil differential metabolites (top 10 VIP value), and bacterial biomarkers (LDA Score > 3.5) following OFBa treatment (Spearman, *p* < 0.05). The solid line indicates a positive correlation and the dashed line indicates a negative correlation.

As can be seen from the above data, the application of organic and microbial fertilizers promoted bamboo growth and development by reshaping the soil microbial community and altering the soil metabolic processes. We next set out to clarify the regulatory relationship between bamboo growth and development, and the enzymatic activity, microbiome composition, and metabolite levels in the soil after fertilization. To this end, we constructed a step-by-step correlation network between the physiological indicators of bamboo growth and the above mentioned soil-related parameters (Figure 8; Supplementary Figure S9). There were four levels in the overall association network, ranging from macroscopic bamboo growth phenotypes to microscopic soil microbial phenotypes. The first level was the plant growth phenotype, which included the soluble sugar and nitrogen content of the leaves, and the lignin and cellulose content of the first internode. The second level was the soil fertility index, which included the activities of four soil enzymes. The third level include data on the top 10 VIP DEMs in the soil (Supplementary Table S6). The fourth level included information on the dominant bacteria associated with each fertilization condition. Using this network, we could clearly find the stepwise regulatory relationships between the four levels.

Among the fertilization treatment groups, only the OFBa group had the closest global stepwise regulatory network (Figure 8). We found that the soluble sugar content of bamboo leaves was positively correlated with urease activity (as a soil fertility indicator). Moreover, urease activity was positively correlated with tropinone and diisobutyl phthalate levels, and negatively correlated with D-maltose levels. Tropinone levels were also positively correlated with *g_Bradyrhizobium*, *o_Frankiales*, the dominant soil microbial signatures. Diisobutyl phthalate levels were positively correlated with the *o_Solirubacterales*, *o_Bacillales*, *g_Bacillus* and *o_Frankiales* signatures. Nitrogen content in bamboo leaves was negatively correlated with acid phosphatase activity, which in turn was positively correlated with the levels of soil metabolites D-maltose and quinoline 3-carboxamides. After OFBa fertilization, the concentrations of D-maltose and quinoline 3-carboxamides were significantly reduced, and were negatively correlated with 9 and 10 of the 15 dominant soil microorganisms, respectively. It is possible that the increase in the abundance of these dominant microorganisms further alters soil fertility by regulating soil metabolic processes in favor of bamboo growth. In addition, we also found relationships between the components within each level. For instance, in the OFBa treatment group, the dominant microflora were more closely correlated with each other, suggesting that these microorganisms participated in the regulation of soil fertility in a synergistic manner to promote bamboo growth.

## 4. Discussion

In this study, we used high-throughput sequencing of 16S rRNA amplicons and LC–MS to characterize the microbiome and metabolome of soil treated with microbial fertilizers, organic fertilizers, or their combinations and assessed the impact on *D. farinosus* growth. Changes in bacterial community structure, abundance index, and metabolite levels were observed in the presence of various fertilizers. Organisms that constitute the soil edaphon form complex structural networks to regulate nutrient cycling, plant growth, ecosystem stability, and sustainability (Zhang et al., 2019). The replenishment of soil nutrients by fertilization inevitably impacts the structure of the soil microbial community (Ling et al., 2017). For instance, the addition of microbial or organic fertilizer to soil remarkably altered the compositional structure of bacterial species (Tao et al., 2020; Dincă et al., 2022). We found that the effect of biological or organic fertilizers on the microbiome of *D. farinosus* varied considerably, and could be explained by differences in soil nutrient availability.

Soil bacterial communities play essential roles in ecosystem functions and regulate key processes in the soil nutrient cycle (such as carbon and nitrogen metabolism) to promote plant growth (Gans et al., 2005). Our findings demonstrated that fertilization altered the composition of soil bacterial populations, which was evidenced by an increase in the number of OTUs in the soil treated with microbial fertilizers and a decrease in the number of OTUs in the soil treated with organic fertilizer; of note, changes in OTU counts did not overtly affect bacterial diversity (Figures 3B,D–E). This result is similar to previous findings (Javoreková et al., 2015; Talwar et al., 2017; Dong et al., 2019). It is well known that both *B. mucilaginosus* and *B. amyloliquefaciens* increase the availability of phosphorus in the soil (Luo et al., 2022). However, occasionally sufficient soil nutrients can reduce the soil bacterial abundance. This is because organic fertilizers rapidly increased the levels of soil nutrient such as TOC, TP, and TN (Supplementary Table S1), which could directly restrict the growth of oligotrophic bacteria that had adapted to the habitat (Koch, 1997). Previous studies have shown that the composition of the soil microbiome is profoundly shaped by fertilization conditions (Dincă et al., 2022), with is in agreement with the finding of the present study (Figure 4; Supplementary Figures S3, S4). Notably, the addition of microbial and organic fertilization directly or indirectly alters the soil nutrient status and may be correlated with soil bacterial community composition by increasing or decreasing the numbers specific bacteria (Zhao et al., 2016; Liang et al., 2020). In line with this view, we found that BmK and Ba treatments increased the relative abundance of *Becteroidota* (16.27 and 13.01%, respectively), but decreased the relative abundance of *Acidobacteriota* (8.63 and 8.15%, respectively) and *Gemmatimonadota* (5.97 and 6.95%, respectively). Different indigenous bacteria respond differently to exogenous substances. Moreover, competition among bacterial species means that while the number of some taxa are enhanced, the abundance of others is inhibited (Marschner et al., 2001; Castro-Sowinski et al., 2007; Mącik et al., 2020). We observed that among the top 10 most abundant phyla, the relative abundances of *Acidobacteriota* (8.31 and 8.61%, respectively), *Myxococcota* (1.96 and 2.88%, respectively), *Fibrobacterota* (0.07 and 0.06%, respectively), and *Verrucomicrobiota* (0.11 and 0.14%, respectively) were significantly reduced, while the relative abundance of *Actinobacteriota* (20.03 and 19.64%, respectively) was significantly increased under OFBa and OFBmK treatment conditions (Figure 4). Notably, the bacterial populations that declined following fertilization with OFBa and OFBmK were oligotrophic species, which could explain the drop in the number of these bacteria after fertilization. However, another study showed that the abundance of some oligotrophic bacteria such as *Acidobacteria* was significantly increased by the application of organic fertilizer (Wang et al., 2016). Consequently, these changes in bacterial composition may profoundly affect the metabolic products of soils and plants.

The importance of soil microorganisms in soil metabolic activity cannot be ignored (Kalu et al., 2022). In addition, the variations in microbial types and their abundance shape the levels and types of soil metabolites, and, in turn, the circulation and metabolism of exogenous nutrients in the soil (Cheng et al., 2022). Uncovering the relationships between soil metabolism and bacterial communities is therefore highly critical to understanding how fertilization can positively affect crop yield without inducing harmful side-effects. A previous study demonstrated a negative correlation between a phylum of thick-walled *Actinomyces* OTUs and the accumulation of uridine, L-DOPA, asymmetric dimethylglycine, adenosine, and phenylalanine in the soil (Kalu et al., 2022). In addition, another study confirmed that different bacterial groups, which regulate starch, sucrose, and citric acid metabolism in the soil, induced different soil metabolic profiles (Song et al., 2020). Our results revealed that there was a significant correlation between the dominant species of soil bacteria and key metabolites involved in multiple metabolic processes following fertilization. This also supports the idea that soil microorganisms can promote or inhibit the accumulation of soil metabolites (Rodríguez et al., 2020). Additionally, we found that fertilizer type was closely related to the structure and complexity of the soil microbial network (Figure 8; Supplementary Figure S9). This may be related to the suppressive effect of soil on plant diseases (Semenov et al., 2021). For instance, *Streptomyces* acts as a bacterial antagonist of plant pathogens in the soil (Mahmoudi et al., 2011; Couillerot et al., 2014). Therefore, in the soil-microbe-plant system, there may be an optimal soil microbial community, which promotes plant growth and protects against disease (Sturz and Christie, 2003). Moreover, the close relationship between specific bacterial species and soil metabolites could guide us to optimize the soil-microbe-plant system through agricultural management or biotechnology.

Soil metabolites are mainly produced by plant roots and edaphic microorganisms. The composition of root secretions varies according to plant species, genetics, and environmental stresses (Zhang et al., 2014; Jian et al., 2016; Li et al., 2020). Differences in soil metabolite composition and abundance can shed light on how soil microorganisms respond to soil nutrients, either directly or indirectly (Li et al., 2019). Our results showed that organic and microbial fertilizers significantly affected soil metabolites, particularly lipids and lipid-like molecules, organic acids, derivatives, organic oxygen compounds, and organoheterocyclic compounds (Figure 6D; Supplementary Figure S6), which in turn interfered with certain pathways involved in galactose, amino acid, and nucleotide metabolism (Figure 8; Supplementary Figure S8). In addition, we observed significant changes in the metabolites involved in these metabolic pathways, such as guanine, which is involved in nucleotide synthesis. Nucleotides are substrates for RNA and DNA synthesis and serve as the main energy source for cellular processes (Kilstrup et al., 2005). Our results showed that organic fertilizers (OF, OFBa, and OFBmK) significantly inhibited the synthesis of nucleotide metabolites in the soil. In accordance with this, we found that the levels of hypoxanthine, deoxycytidine, deoxyguanosine, and cytosine, all of which perform essential roles in nucleotide metabolism, were significantly reduced after organic fertilization (Tomoike et al., 2015). Galactitol is a natural product found in organisms such as *Elaeodendron croceum* and *Salacia chinensis* (Adriano Rutz, 2022). It is also used as a fermentation substrate by numerous species of bacteria such as *Pseudomonas* and *Clostridium* (Rahman Khan et al., 1992; Jeon et al., 2013). The addition of organic fertilizers (OF, OFBa, or OFBmK) significantly decreased the metabolism of galactitol *via* the galactose metabolic pathway and PTS in our study. PTS is a multifaceted system required for carbohydrate uptake, carbon catabolite metabolite inhibition, and chemotaxis (Postma et al., 1993). In addition, links between carbon metabolism and other cellular processes have been reported, for example, *via* nitrogen fixation by PTS, the stress response, starvation, and pathogenicity (Lodget and Jacobson, 1988; Rabus et al., 1999; Gaurivaud et al., 2000; Ueguchi et al., 2001). Similarly, OF significantly reduced the synthesis of L-glutamate, which is not only a precursor of glutamine, proline, arginine, and lysine, but also a participant in the urea cycle (Wang and Topputo, 2020). Meanwhile, we found that the levels of L-glutamate-related metabolites (2-oxoarginine, anserine, and γ-glutamyl-β-aminopropiononitrile) were significantly increased (25.67-, 13.80-, and 25.04-fold, respectively, versus the control group). Furthermore, the levels of the DEM xylobiose were significantly increased in our study (7.94-, 6.26-, and 6.31-fold, respectively, versus the control), presumably due to the elevated relative abundance of *Streptomyces* (Figure 4F). The xylan-degrading enzymes have been extensively reported in *Streptomyces* species such as *Streptomyces matensis* DW67, *Streptomyces* sp. strain S38, *Streptomyces cyaneus* SN32, *Streptomyces olivaceoviridis* E-86, *Streptomyces actuosus* A-151, and *Streptomyces rameus* L2001 (Simkhada et al., 2012). Among these enzymes, endoxylanase can degrade xylan into xylobiose and xylotriose (Li et al., 2012). Collectively, these results support the idea that organic fertilizers (OF, OFBa, and OFBmK) cause stronger and more complex perturbations to the metabolism of soil plants and microorganisms, compared to microbial fertilizers (Ba and BmK).

Moreover, our findings revealed the interaction between soil metabolites and bacterial communities. Correlation analysis showed that the activities of soil urease activity and acid phosphatase were significantly correlated with the levels of metabolites such as D-maltose and quinoline-3-carboxamides under OFBa and OFBmK treatment conditions (Figure 8; Supplementary Figure S9). Acid phosphatase activity directly affects the catabolic transformation of soil organic phosphorus and its bioefficacy (Wang et al., 2018), while soil urease activity is significantly correlated with the ability of microorganisms to utilize soil nitrogen (Tao et al., 2018). It is possible that *B. mucilaginosus* and *B. amyloliquefaciens*, which are nourished by organic fertilizers, drive a catabolic transformation of soil nitrogen and phosphorus, which in turn promotes plant growth. The significant increase in soil AN and AP and *D. farinosus* phenotypic analysis confirmed this view (Figure 2; Supplementary Table S1).

Despite the fact that our metabolomic studies are based on specific conditions and time points, it would be of great interest to establish a link between changes in bacterial community structure and metabolites in the soil over time and/or space. Thus, the present findings still leave much to be desired, lacking temporal and spatial evidence in realistic scenarios. The results of our experiments only illustrate the effects of short-term fertilization. Thus, the long-term mechanisms and ongoing effects of organic and microbial fertilization on the soil microbiome and metabolome remain to be investigated. In addition, soil bacteria coexist in complex ways with other organisms. Therefore, investigating the mechanisms of how bacteria interact with their complex environment to improve bamboo growth in various soil-*D. farinosus* scenarios through a multi-omics combination will be a fascinating topic for future research.

## 5. Conclusion

Our results confirm that changes in the soil bacterial community composition, and consequently the types and levels of soil metabolites, are related to the addition of microbial and organic fertilizers in a greenhouse experiment involving potted *D. farinosus* plants. Both microbial and organic fertilizers significantly altered bacterial composition and disturbed bacterial symbiotic networks. Metabolomics results revealed that treatment with microbial and organic fertilizers altered soil metabolism, which was related to the changes of certain in specific bacterial taxa and their enzymatic activities. Altogether, the results of the present study highlight that a better understanding of how fertilizers regulate the biological attributes of soil bacteria to improve the quality of the soil is crucial for advancing agricultural practices in an environmentally sustainable manner.

## Data availability statement

The raw 16S rRNA gene amplicon sequences supporting for this study have been deposited in the National Center for Biotechnology Information (NCBI) database under the BioProject accession number PRJNA926013.

## Author contributions

SHu and YC designed the research. SL, SHa, BD, and SR performed the research. WF, SL, XZ, and SHu analyzed the data. WF, GX, and SHu wrote the manuscript. All authors contributed to the article and approved the submitted version.

## Funding

This work was supported by grant from National Key R&D Program of China (2021YFD2200504_1 and 2021YFD2200505_2), the Science and Technology Project of Sichuan Province, China (2022NSFSC0093 and 2021YFYZ0006), and Ph.D. Foundation (no. 22zx7146) from Southwest University of Science and Technology.

## Conflict of interest

The authors declare that the research was conducted in the absence of any commercial or financial relationships that could be construed as a potential conflict of interest.

## Publisher’s note

All claims expressed in this article are solely those of the authors and do not necessarily represent those of their affiliated organizations, or those of the publisher, the editors and the reviewers. Any product that may be evaluated in this article, or claim that may be made by its manufacturer, is not guaranteed or endorsed by the publisher.

## Footnotes

^1^ https://www.kegg.jp/

^2^ https://www.omicshare.com/tools/
