# Supplementary material for: Bio-organic fertilizers improve Dendrocalamus farinosus growth by remolding the soil microbiome and metabolome
Source: Front Microbiol. 2023 Feb 15;14:1117355. doi: 10.3389/fmicb.2023.1117355 (PMC9975161; doi:10.3389/fmicb.2023.1117355)
Supplement: Supplementary file 6 [file Presentation_1.PDF]

Supplementary Material

Bio-organic fertilizers improve *Dendrocalamus farinosus* growth by remolding the soil microbiome and metabolome

Shangmeng Li[1#], Wei Fan[1#], Gang Xu, Ying Cao, Xin Zhao, Suwei Hao, Bin Deng, Shanglian Hu[1*]

*** Correspondence:** Corresponding Author: hushanglian@swust.edu.cn

1 Supplementary Figures and Tables

1.1 Supplementary Figures

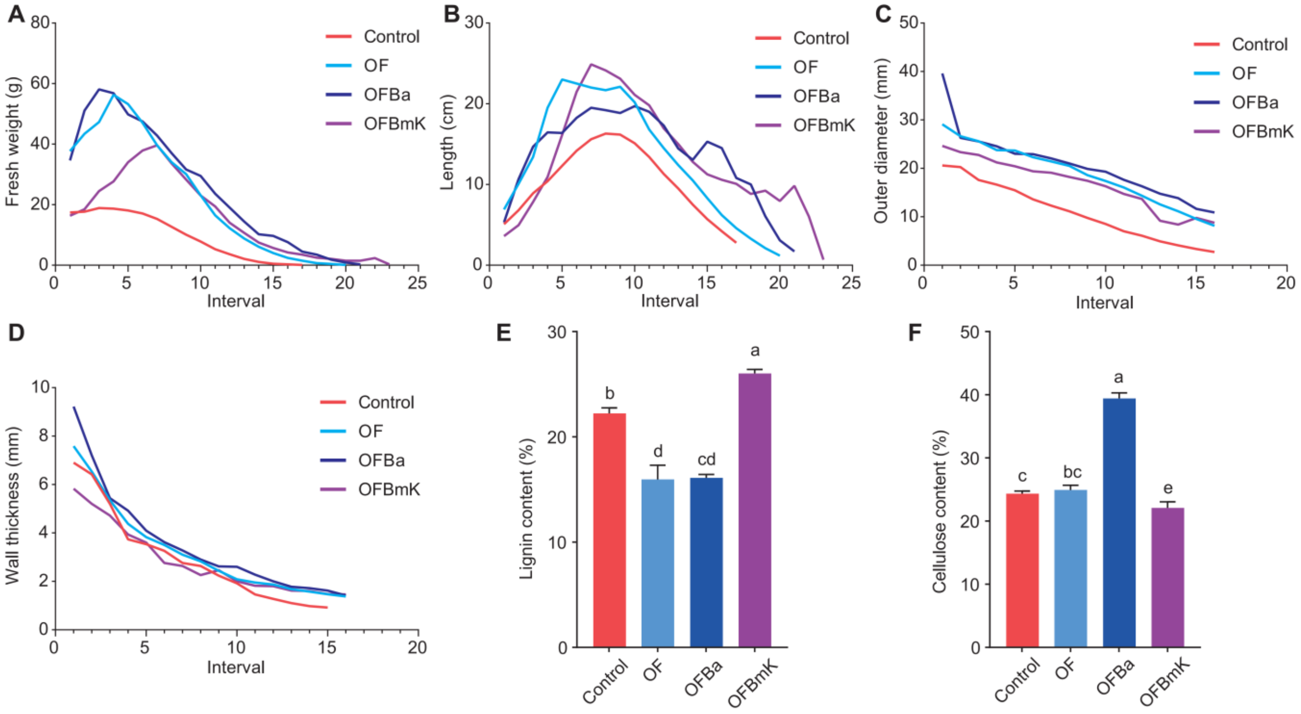

Supplementary Figure 1. Analysis of fresh weight **(A)**, length **(B)**, diameter **(C)** and wall thickness **(D)** of different internodes of *Dendrocalamus farinosus*, and the lignin **(E)** and cellulose **(F)** content of the first internode of new shoots. OF, organic fertilizer; OFBa, bio-organic fertilizer (containing *Bacillus amyloliquefaciens*); OFBmK, bio-organic fertilizer (containing *Bacillus mucilaginosus Krassilnikov*).

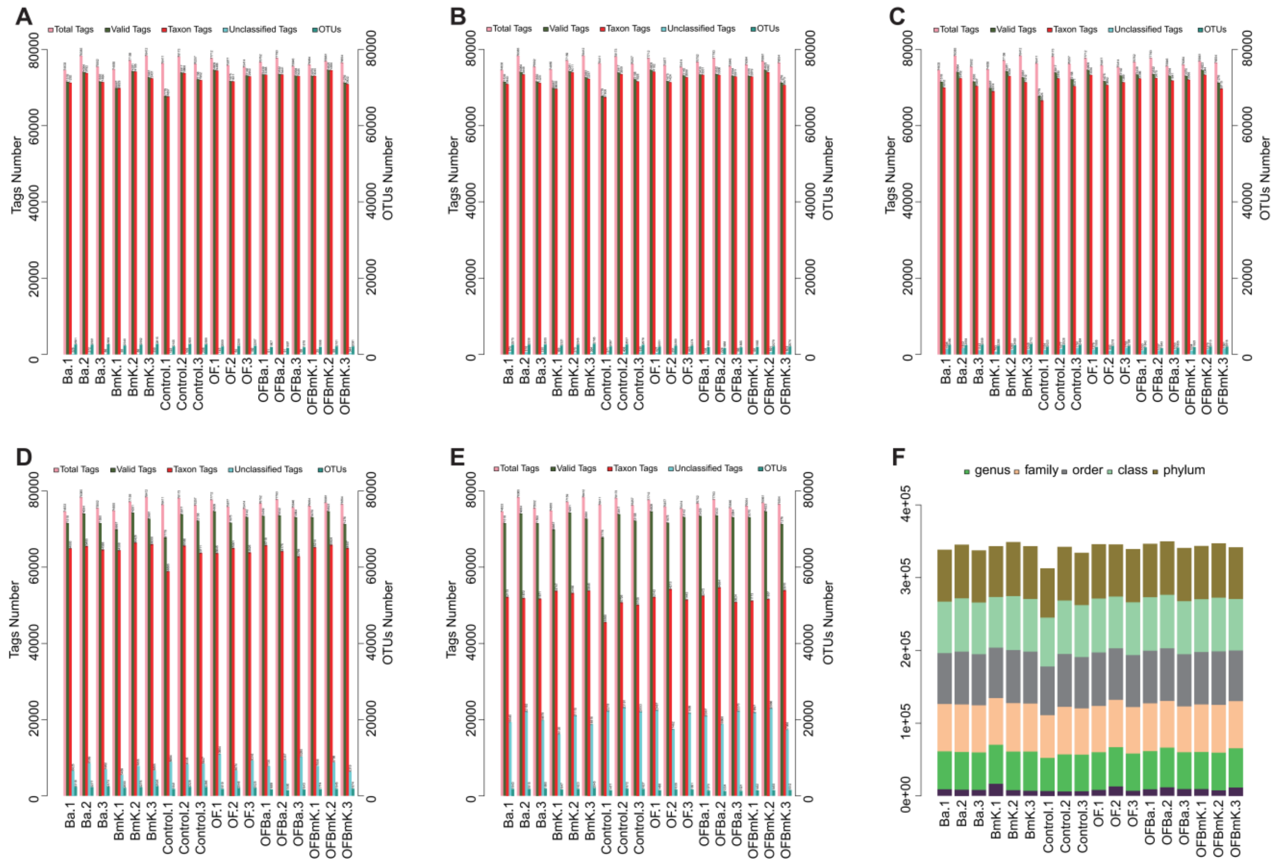

Supplementary Figure 2. Operational taxonomic units (OTUs) classified according to phylum **(A)**, class **(B)**, order **(C)**, family **(D)**, and genus **(E)** levels in each treatment group. Results of the statistical analysis of total tags, valid tags, and the distributions of valid tags (Taxon Tags) that were used to build OTUs and obtain annotated information and valid tags that did not carry annotated information (Unclassified Tags) for each treatment condition. Statistical analysis of the total number of OTU tags associated with different classification levels **(F)**.

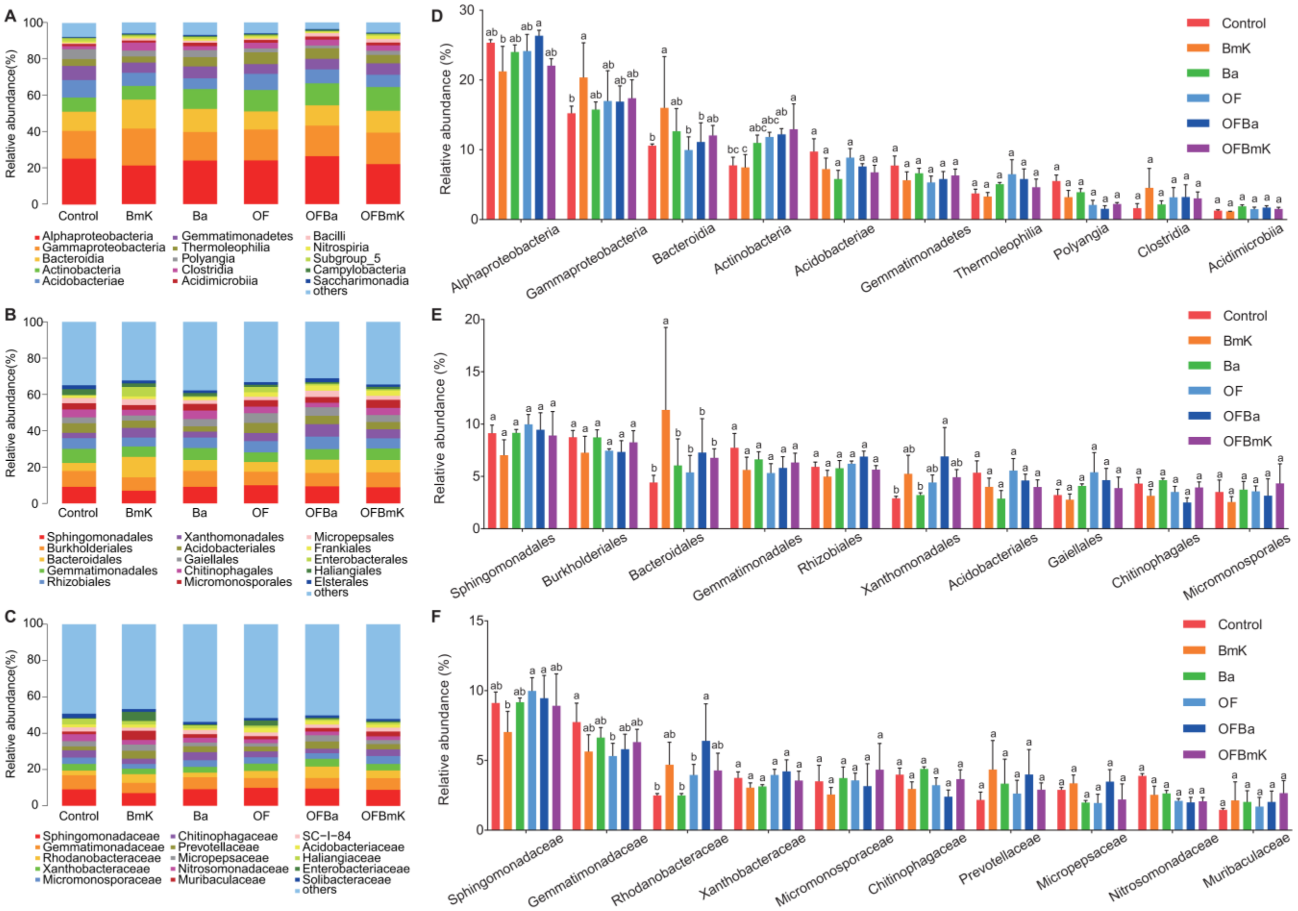

Supplementary Figure 3. Microbial community structure analysis at the class, order, and family levels. Histogram of the top 15 relatively abundant bacteria at the class **(A)**, order **(B)**, and family **(C)** levels. The top 10 most abundant bacterial species at the class **(D)**, order **(E)**, and family **(F)** levels were selected and the differences between treatments groups were calculated using the one-way ANOVA and Tukey post-hoc test; different letters represent significant differences between the mean values ($p < 0.05$, $n = 3$).

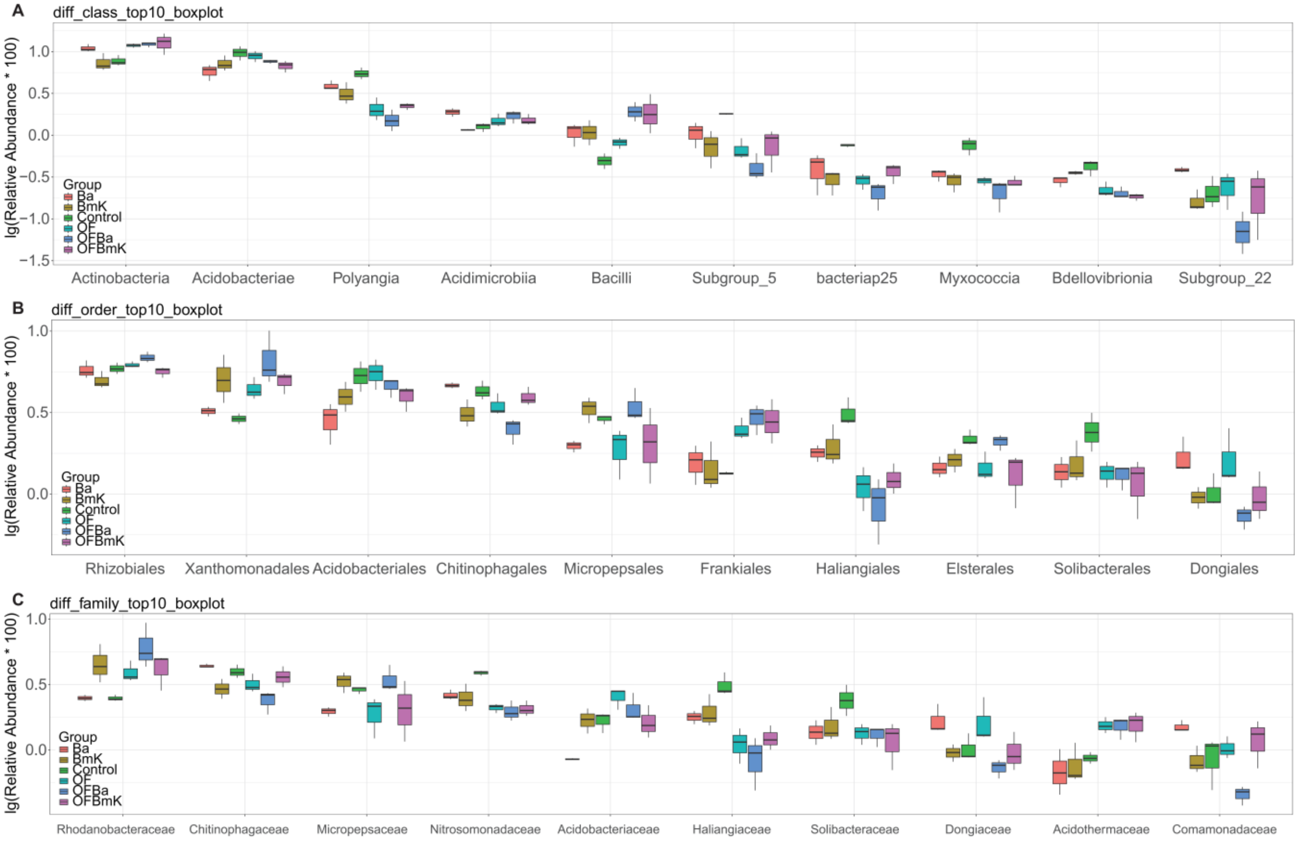

Supplementary Figure 4. Boxplot of differential species at the class **(A)**, order **(B)**, and family **(C)** levels. The top 10 diverse species were selected and analyzed by ANOVA to compare the abundance of dominant species within and between treatment groups.

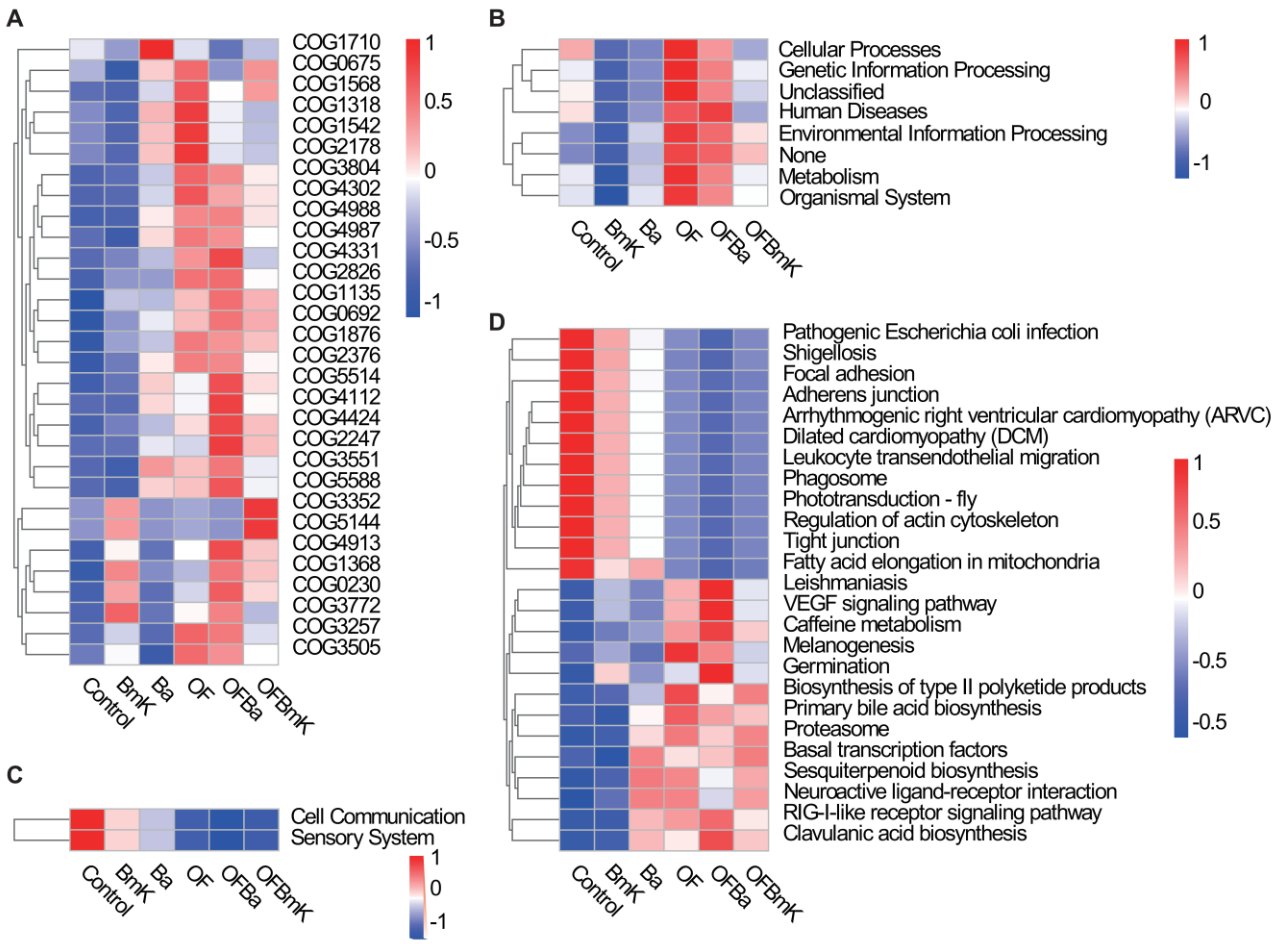

Supplementary Figure 5. The prediction of COG and KEGG functions based on 16S rRNA sequences. The predicted homologous protein cluster (COG) and KEGG results were statistically tested using the Kruskal–Wallis algorithm. The results showed a total of 555 differential COGs **(A)**. 0, 2, and 25 metabolic pathways at KEGG_level L1 **(B)**, L2 **(C)**, and L3 **(D)**, respectively, were significantly different. Detailed annotations of the KEGG and COG analyses of 16S rRNA sequences are shown in Supplementary Table 3.

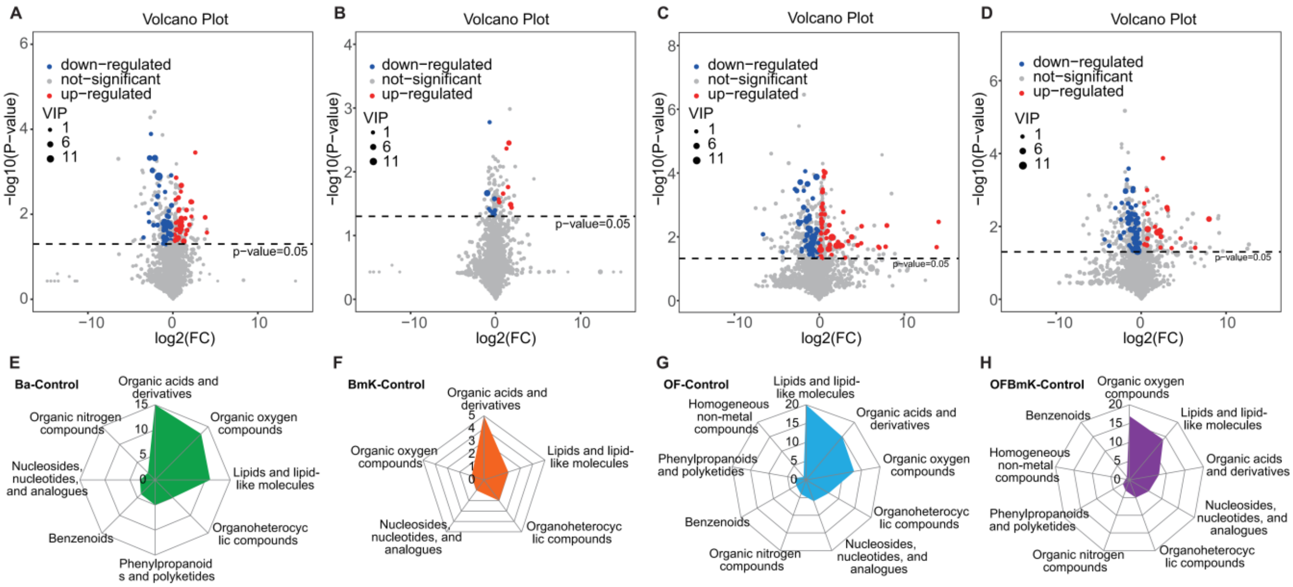

Supplementary Figure 6. Classification statistics of soil differential metabolites in the Ba **(A)**, BmK **(B)**, OF **(C)**, and OFBmK **(D)** treatment groups.

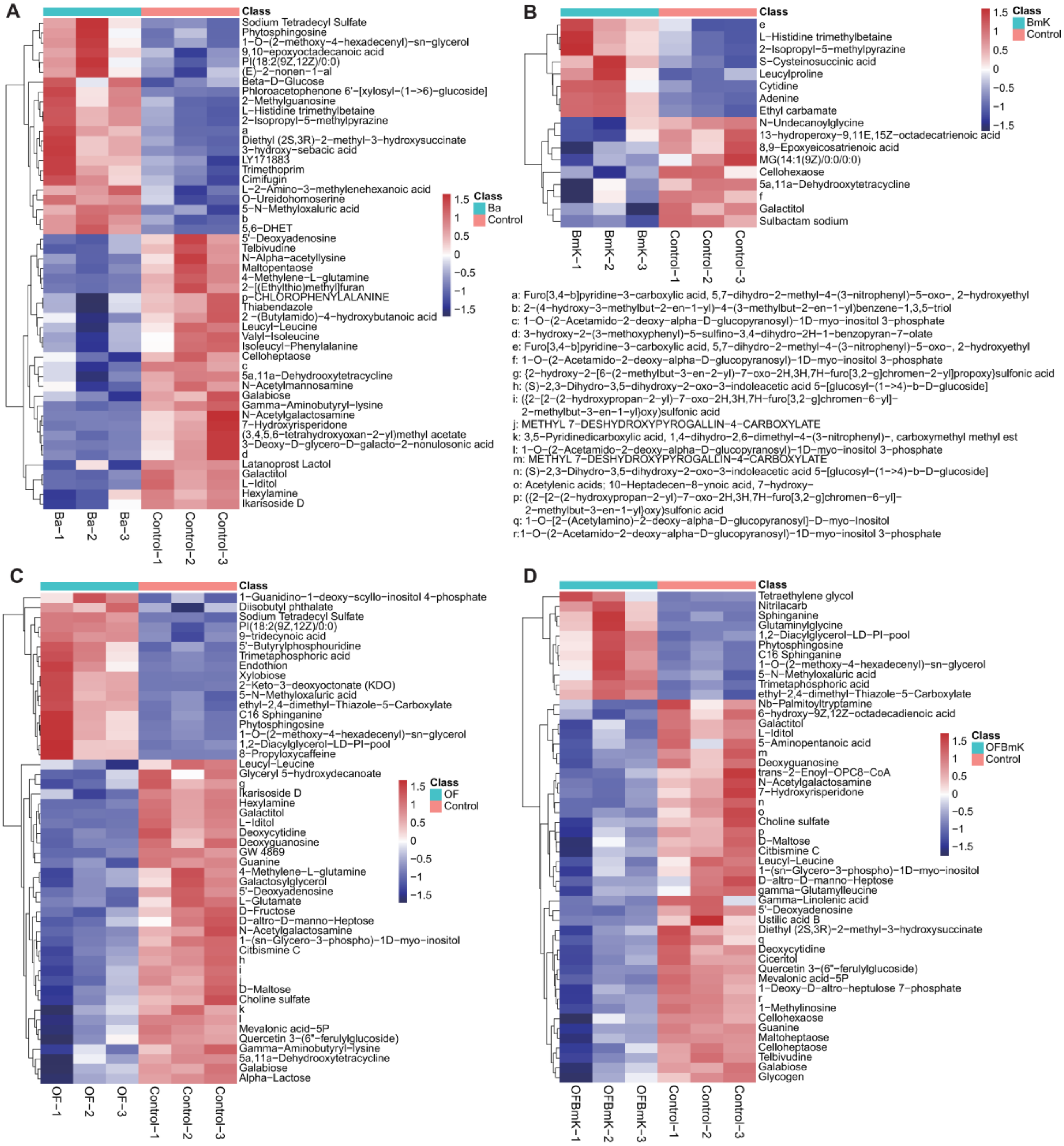

Supplementary Figure 7. The fold changes in differential metabolite (top 50) content among the Ba **(A)**, BmK **(B)**, OF **(C)**, and OFBmK **(D)** treatment groups.

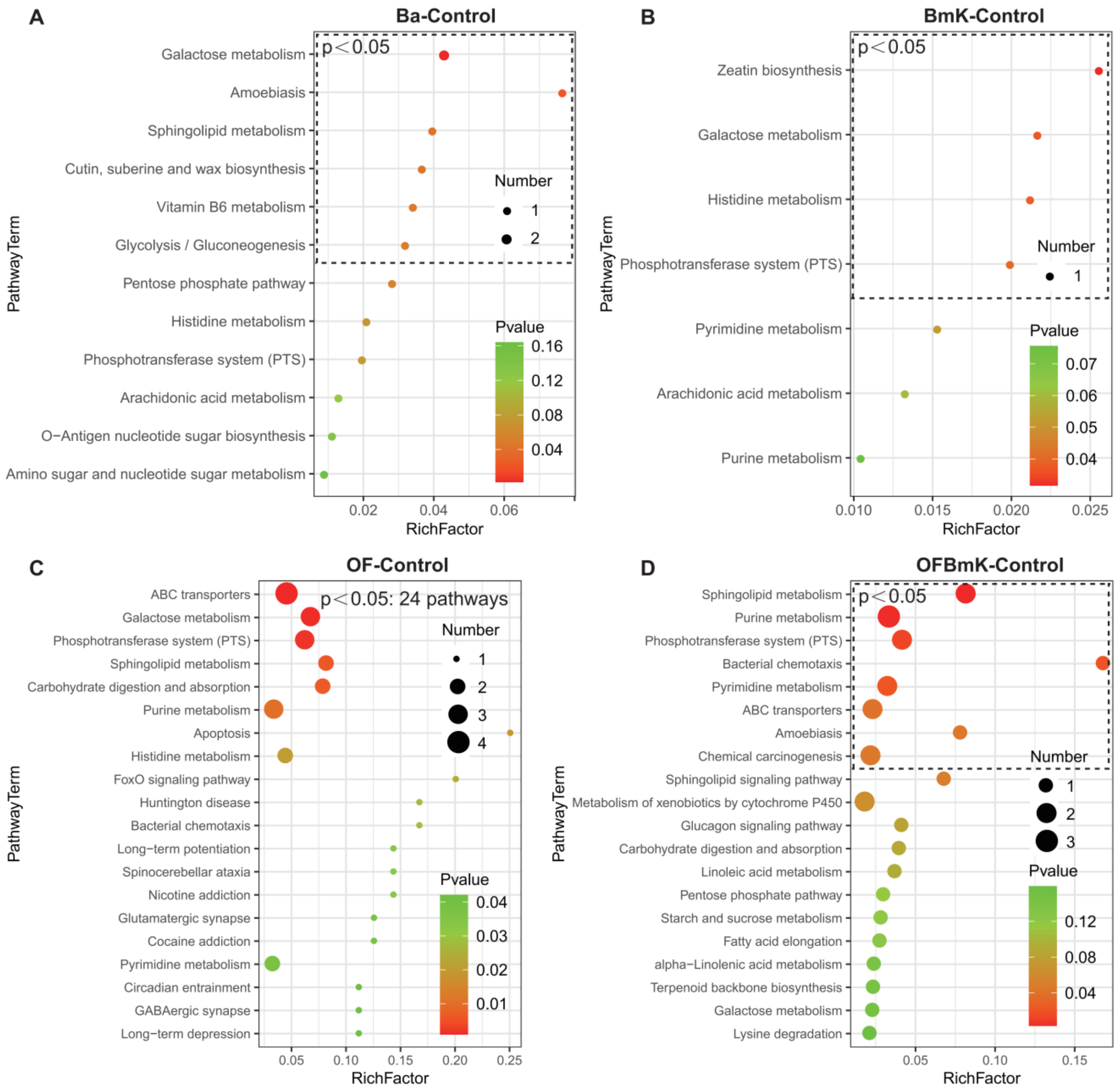

Supplementary Figure 8. Enrichment analysis of differential-metabolite-associated metabolic pathways (top 20) among the Ba **(A)**, BmK **(B)**, OF **(C)**, and OFBmK **(D)** treatment groups.

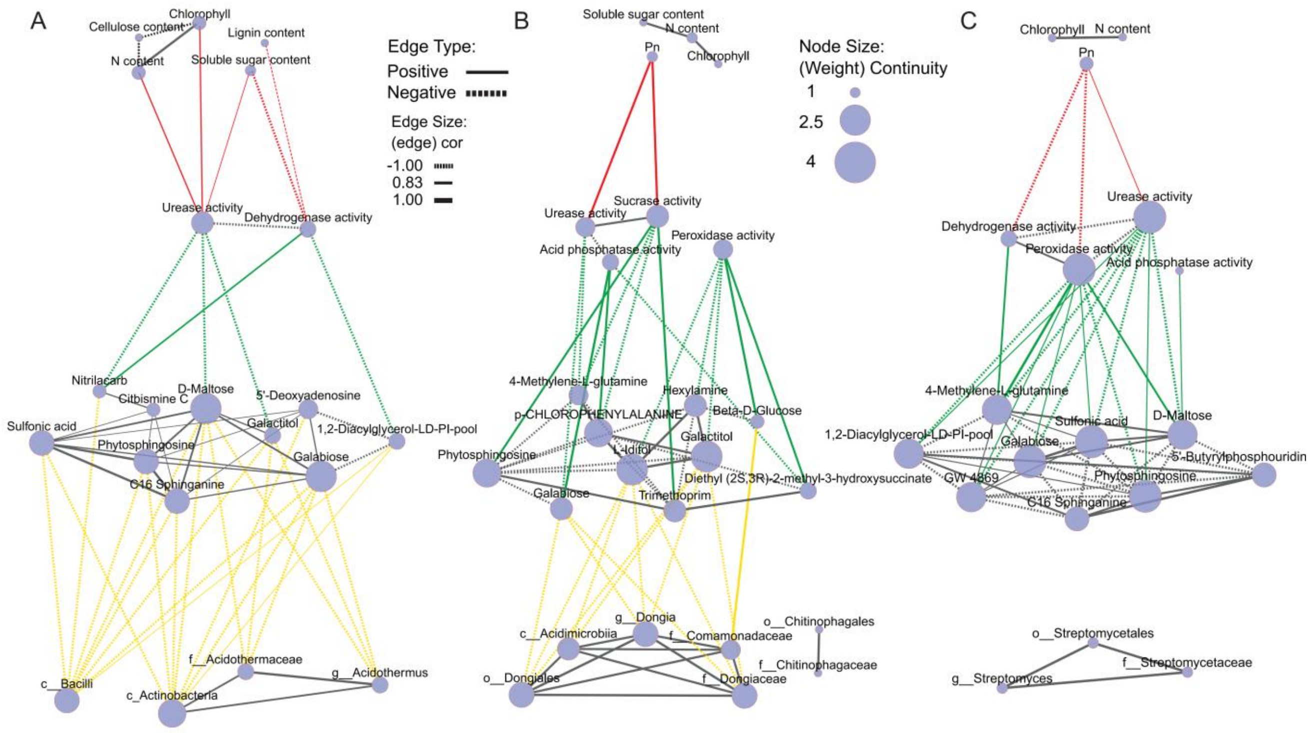

Supplementary Figure 9. Correlation network of *Dendrocalamus farinosus* growth phenotype, soil enzymatic activity, soil differential metabolite levels (top 10 VIP value), and bacterial biomarkers (LDA Score > 3.5) among the OFBmk **(A)**, Ba **(B)**, and OF **(C)** treatment groups (Spearman, $p < 0.05$). The solid line indicates a positive correlation and the dashed line indicates a negative correlation.

1.2 Supplementary Tables

Supplementary Table 1. Soil chemical properties following different fertilization regimens

Treatment	CK	BmK	Ba	OF	OFBa	OFBmK
TC (g kg^{-1})	2580.12 ± 99.99c	2168.91 ± 100.39d	1956.57 ± 99.40e	2774.81 ± 99.03b	2859.14 ± 100.04b	3516.48 ± 99.53a
TOC (g kg^{-1})	2761.08 ± 99.26b	2405.24 ± 100.50c	2064.78 ± 98.68d	2858.66 ± 100.74b	3668.12 ± 99.18a	3609.33 ± 100.06a
TN (g kg^{-1})	193.01 ± 5.50ab	154.89 ± 5.63c	142.78 ± 5.46c	185.31 ± 5.07b	194.35 ± 5.07ab	216.99 ± 5.53a
TP (mg kg^{-1})	150.94 ± 15.56e	609.95 ± 15.40d	868.89 ± 15.72a	589.94 ± 15.40d	696.03 ± 15.46b	636.59 ± 15.07c
AN (mg kg^{-1})	206.55 ± 5.06bc	184.20 ± 4.80cd	164.65 ± 4.92d	212..26 ± 5.12b	259.91 ± 5.05a	226.11 ± 5.43b

All of the values for potted soil samples were reported as the means ± standard error (n = 3). Different letters in the same line indicate a significant difference at $P < 0.05$ according to Tukey's test. TC, total carbon; TOC, total organic carbon; TN, total nitrogen; TP, total phosphorus; AN, avaliable nitrogen. BmK, *Bacillus mucilaginosus Krassilnikov*; Ba, *Bacillus amyloliquefaciens*; OF, organic fertilizer; OFBa, bio-organic fertilizer (containing *Bacillus amyloliquefaciens*); OFBmK, bio-organic fertilizer (containing *Bacillus mucilaginosus Krassilnikov*).

Supplementary Table 2. Detailed operational taxonomic unit count data (see separate Excel file)

Supplementary Table 3. Detailed data from the COG and KEGG analyses of 16S rRNA sequences (see separate Excel file)

Supplementary Table 4. Detailed data for all soil metabolites analyzed (see separate Excel file)

Supplementary Table 5. Detailed data for all soil metabolic pathway enriched following fertilization (see separate Excel file)

Supplementary Table 6. Fold changes in the differential metabolites with the top 10 VIP values in the soil (see separate Excel file)
